# MAIT and other innate-like T cells integrate adaptive immune responses to modulate interval-dependent reactogenicity to mRNA vaccines

**DOI:** 10.1126/sciimmunol.adu3337

**Published:** 2025-08-29

**Authors:** Ali Amini, Lucy C. Garner, Robert H. Shaw, Neil Wrigley Kelly, Sandra Adele, Donal T. Skelly, Wanwisa Dejnirattisai, Melanie Greenland, Xinxue Liu, Amelia Heslington, Carl-Philipp Hackstein, Sam M. Murray, Cristina Riquelme Vano, Lizzie Stafford, Sile Johnson, Katia Sayaf, Maria Fransiska Pudjohartono, Elizabeth A. Clutterbuck, PITCH Consortium, PMohammad Ali, Alice Bridges-Webb, Jeremy Chalk, Alexandra S. Deeks, Christina Dold, David Eyre, John Frater, Lisa Frending, Philip Goulder, Anni Jamsen, Tom Malone, Philippa C. Matthews, Eloise Phillips, Patpong Rongkard, Beatrice Simmons, Lance Turtle, Com-COV Study Group, PArabella S. V. Stuart, Parvinder K. Aley, Nick J. Andrews, J. Claire Cameron, Sue Charlton, Andrea Collins, Tanya Dinesh, Anna England, Saul N. Faust, Daniela Ferreira, Adam Finn, Christopher A. Green, Bassam Hallis, Paul Heath, Helen Hill, Rajeka Lazarus, Vincenzo Libri, Yama Mujadidi, Emma Plesteda, Mary Ramsay, Robert C. Read, Hannah Robinson, Nisha Singh, David P. J. Turner, Paul J. Turner, Rachel White, Jonathan S. Nguyen-Van-Tam, Sagida Bibi, Christopher P. Conlon, Tim James, Katie Jeffery, Barbara Kronsteiner, Alexander J. Mentzer, Donal O’Shea, Maheshi N. Ramasamy, Gavin R. Screaton, Matthew D. Snape, Andrew E. Hogan, Eleanor Barnes, Teresa Lambe, Susanna J. Dunachie, Nicholas M. Provine, Paul Klenerman

**Affiliations:** 1 Translational Gastroenterology and Liver Unit, Nuffield Department of Medicine – Experimental Medicine, University of Oxford, Oxford, UK; 2 Peter Medawar Building for Pathogen Research, Nuffield Department of Medicine, University of Oxford, Oxford, UK; 3 Pandemic Sciences Institute, Nuffield Department of Medicine, University of Oxford, Oxford, UK; 4 Oxford University Hospitals NHS Foundation Trust, Oxford, UK; 5 Oxford Vaccine Group, Department of Paediatrics, University of Oxford, Oxford, UK; 6 St Vincent’s University Hospital & University College Dublin, Dublin, Ireland; 7 Nuffield Department of Clinical Neurosciences, University of Oxford, Oxford, UK; 8 Centre for Human Genetics, Nuffield Department of Medicine, University of Oxford, Oxford, UK; 9 Division of Emerging Infectious Disease, Research Department, Faculty of Medicine Siriraj Hospital, Mahidol University, Bangkok Noi, Bangkok 10700, Thailand; 10 Center for Infection Prevention, Technical University of Munich, Munich, Germany; 11 Nuffield Department of Medicine, University of Oxford, Oxford, UK; 12 Radcliffe Department of Medicine, University of Oxford, Oxford, UK; 13 NDM Centre for Global Health Research, Nuffield Department of Medicine, University of Oxford, Oxford, UK; 14 Kathleen Lonsdale Institute for Human Health Research, Maynooth University, Maynooth, Ireland; 15 NIHR Oxford Biomedical Research Centre, Oxford University Hospitals NHS Foundation Trust, Oxford, UK; 16 Chinese Academy of Medical Science (CAMS) Oxford Institute (COI), University of Oxford, Oxford, UK; 17 National Children’s Research Centre, Dublin, Ireland; 18 Mahidol-Oxford Tropical Medicine Research Unit, Mahidol University, Bangkok, Thailand; 19 Big Data Institute, Nuffield Department of Population Health, University of Oxford, Oxford, UK; 20 NIHR Health Protection Research Unit in Emerging and Zoonotic Infections, Institute of Infection, Veterinary and Ecological Sciences, University of Liverpool, Liverpool, UK; 21 Immunisation and Vaccine Preventable Diseases Division, UK Health Security Agency, London, UK; 22 Health Protection Scotland, Glasgow, UK; 23 UK Health Security Agency, Porton Down, Salisbury, UK; 24 Liverpool School of Tropical Medicine, Liverpool, UK; 25 Faculty of Medicine and Institute for Life Sciences, University of Southampton, Southampton, UK; 26 Bristol Vaccine Centre, Schools of Population Health Sciences and of Cellular and Molecular Medicine, University of Bristol, Bristol, UK; 27 NIHR/Wellcome Trust Clinical Research Facility, University Hospitals Birmingham NHS Foundation Trust, Birmingham, UK; 28 The Vaccine Institute, St George’s University of London, London, UK; 29 NIHR UCLH Clinical Research Facility and NIHR UCLH Biomedical Research Centre, University College London Hospitals NHS Foundation Trust, London, UK; 30 University of Nottingham, Nottingham, UK; 31 National Heart and Lung Institute, Imperial College London, London, UK

## Abstract

Adenoviral (Ad) vectors and mRNA vaccines exhibit distinct patterns of immune responses and reactogenicity, but underpinning mechanisms remain unclear. We longitudinally compared homologous ChAdOx1 nCoV-19 and BNT162b2 vaccination, focusing on cytokine-responsive innate-like lymphocytes – mucosal-associated invariant T (MAIT) cells and Vδ2^+^ γδ T cells – which sense and tune innate-adaptive crosstalk. Ad priming elicited robust type I interferon (IFN)-mediated innate-like T cell activation, augmenting T cell responses (innate-to-adaptive signaling), which was dampened at boost by anti-vector immunity. Conversely, mRNA boosting enhanced innate-like responses, driven by prime-induced spike-specific memory T cell-derived IFN-γ (adaptive-to-innate signaling). Extending the dosing interval dampened inflammation at boost due to waning T cell memory. In a separate vaccine trial, pre-boost spike-specific T cells predicted severe mRNA reactogenicity regardless of the priming platform or interval. Overall, bidirectional innate-like and adaptive crosstalk, and IFN-γ-licensed innate-like T cells, orchestrate interval-dependent early vaccine responses, suggesting modifiable targets for safer, more effective regimens.

## Introduction

The global response to the SARS-CoV-2 pandemic heavily relied on nucleoside-modified mRNA and adenoviral (Ad) vector vaccines. The first major vaccines administered included BNT162b2 (Pfizer/BioNTech, mRNA), mRNA-1273 (Moderna, mRNA), ChAdOx1 nCoV-19 (Oxford-AstraZeneca, chimpanzee-derived Ad vector), and Ad26.COV2-S (Janssen, rare human Ad vector serotype 26). These vaccines induced distinct adaptive immune responses and patterns of systemic reactogenicity after homologous prime-boost regimens, which were influenced by the boosting interval (1–4). For instance, the propensity for myocarditis increased after mRNA vaccine boosting (5–7), whereas Ad vector-induced systemic reactogenicity and vaccine-induced thrombotic thrombocytopenia (VITT) were more pronounced after the initial dose (8–11). Despite these observations and their public health impact, the underlying mechanisms regulating vaccine efficacy and, importantly, tolerability remain to be fully defined.

Both major vaccine platforms employed distinct strategies to manipulate early immune responses, which are crucial for safety and immunogenicity. Ad vector vaccines, such as ChAdOx1 and Ad26, generate specific early cytokines (12, 13), and were selected to avoid the negative implications of pre-existing anti-vector immunity (14, 15). RNA vaccines leveraged nucleoside-modification to evade cytosolic RNA sensors and enhance antigen translation (16, 17). Despite efforts to minimize innate triggering, BNT162b2 induces multiple cytokines after vaccination, with increased levels after boosting that mirror reactogenicity (18–20). Enhanced IFN-γ production after BNT162b2 boosting has been consistently observed (19–22), and is dependent on T cells in mice (23). Early cytokine production and NK cell activation correlate with systemic reactogenicity (20, 22), and have been linked to myopericarditis occurring within days of mRNA boosting (5, 24, 25). Measures to predict and mitigate systemic reactogenicity are limited but include extending the boosting interval (≥ 56 days) (26). Exactly how extended interval boosting modulates early cytokine responses is unknown.

Cytokine-responsive innate-like T cells, particularly mucosal-associated invariant T (MAIT) cells and Vδ2^+^ γδ T cells, are key early effectors in response to ChAdOx1 and are well placed to tune both early inflammatory reactogenicity and immunogenicity (13, 27, 28). MAIT cells are activated early after ChAdOx1 vaccination by type I IFN, with IL-12, IL-18, and TNF (13). Their activation amplifies IFN-γ production and they are required for optimal adaptive CD8^+^ T cell responses (13). MAIT cell IFN-γ, and other IFN-dependent chemokines, are key to protective anti-viral responses and are mechanistically implicated in CD8^+^ T priming (29). This early transcriptional signature of ChAdOx1-induced MAIT cell activation is also found in whole blood following BNT162b2, mRNA-1273, and Ad26 vaccinations, with prolonged activation implicated in pathogenic VITT (30). Baseline MAIT cell characteristics also predict adaptive immune responses to BNT162b2 (31), with transcriptional evidence of activation one day after vaccination (32). Given that nucleoside modification attenuates type I IFN induction, it is unclear how MAIT cell activation might occur following mRNA vaccination. Given their capacity for rapid effector functions and abundance in both human tissues and lymph nodes (33), MAIT cells and other innate and innate-like lymphocytes are well-placed to amplify both protective and pathological early immune responses to major vaccine platforms.

In this study, we longitudinally compared early human immune responses after homologous prime-boost SARS-CoV-2 vaccination with either BNT162b2 or ChAdOx1 nCoV-19, focusing on innate-like lymphocytes. We assessed the impact of boosting interval on BNT162b2 responses, given differences in adaptive immunity and reactogenicity (2, 4). Our findings uncovered shared and unique patterns of innate-like lymphocyte activation ex vivo, which are modulated by adaptive immunity after boosting for both vaccine modalities. Measures of spike-specific adaptive immunity were predictive of systemic reactogenicity following mRNA vaccination and strongly dependent on dosing interval, with implications for vaccine schedules.

## Results

### Longitudinal cohort of immune responses to SARS-CoV-2 vaccines

**Figure 1:**
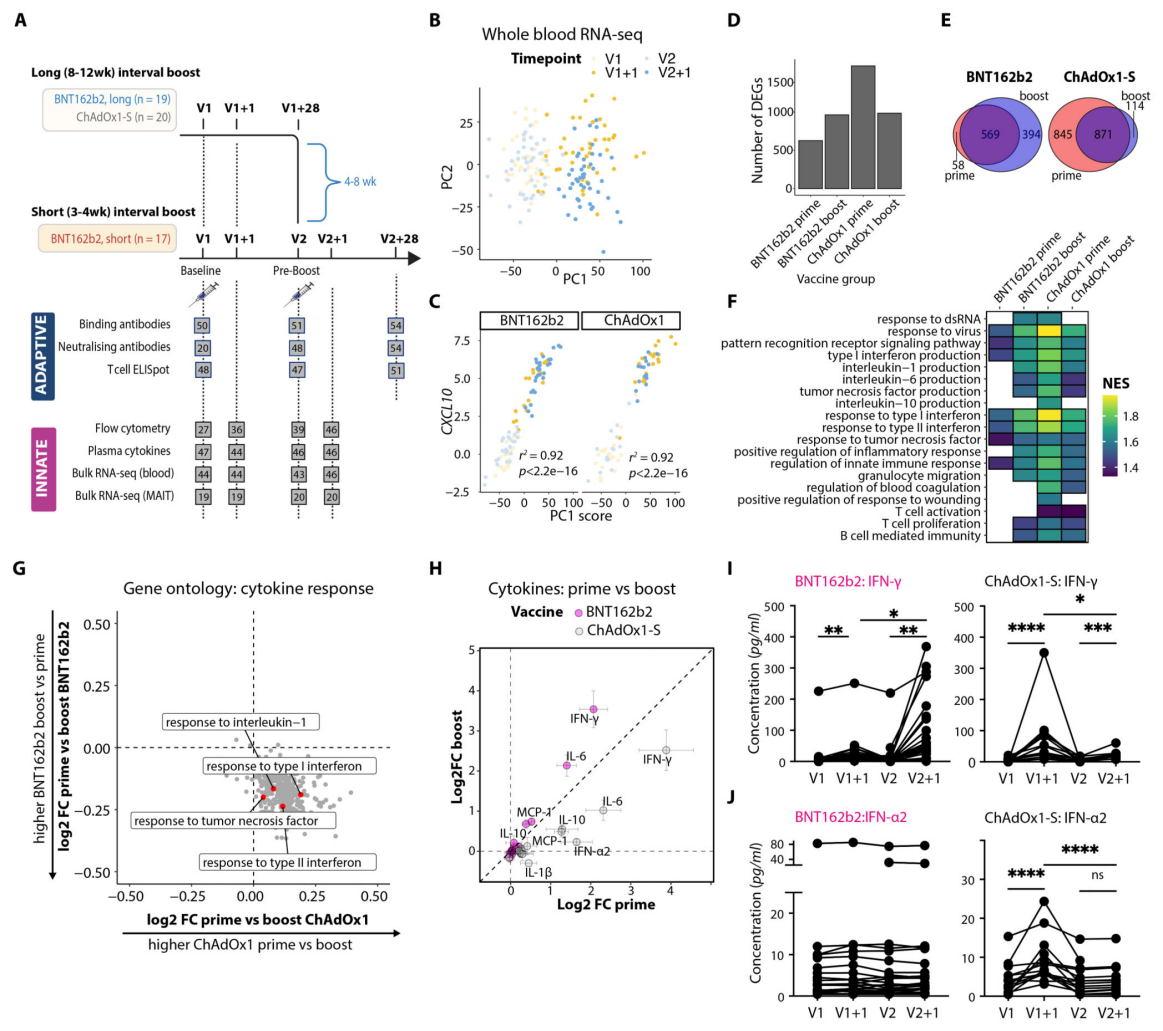
Differential early interferon response to SARS-CoV-2 vaccines. **(A)** Schematic of longitudinal sampling of healthcare workers, including timepoints and assays performed, with grey boxes indicating the number of samples used for each of the assays at the specified timepoints. **(B, C)** Whole blood RNA-seq data, with samples colored by timepoint (pre-prime, V1, and day one post-prime, V1+1, shades of yellow; pre-boost, V2, and V2+1, shades of blue). (B) Principal component analysis (PCA), and (C) correlation between principal component 1 (PC1) scores and *CXCL10* expression. Pearson’s *r*^2^ and p-values are shown. **(D)** Number of differentially expressed genes (DEGs; false discovery rate (FDR) < 0.05, log_2_ fold change > 1) between pre- and post-vaccine timepoints. **(E)** Venn diagrams showing overlapping DEGs at BNT162b2 prime and boost, and ChAdOx1-S prime and boost. **(F)** Gene set enrichment analysis (GSEA) normalized enriched scores (NES) for selected Gene Ontology (GO) Biological Process terms; only significantly enriched terms (FDR < 0.05) are shown. **(G)** Scatter plot of log_2_ fold changes in GSEA NESs for significantly enriched GO terms at prime versus boost for ChAdOx1-S and BNT162b2; labels and red points highlight specific cytokine response pathways. **(H)** Mean log_2_ fold change in plasma cytokines post-prime (V1+1/V1) and post-boost (V2+1/V2) for both vaccines; mean ± SEM shown, with points colored by vaccine group. **(I, J)** Plasma concentrations of (I) IFN-γ and (J) IFN-α2 from vaccinated individuals at specified timepoints; symbols represent individual samples with lines connecting data points from the same donor. Statistical significance determined using mixed-effects ANOVA with Šídák’s correction for multiple comparisons. *p < 0.05; **p < 0.01; ***p < 0.001; ****p < 0.0001; ns, not significant.

We investigated longitudinal early immune responses to SARS-CoV-2 vaccines in 56 healthy adult volunteers and healthcare workers (HCW) receiving their initial homologous prime-boost vaccination with BNT162b2 or ChAdOx1 nCoV-19 (also known as AZD1222; hereafter referred to as ChAdOx1-S). Whole blood, plasma, serum, and peripheral blood mononuclear cells (PBMCs) were collected before and one day following each vaccination dose, and at fixed convalescent timepoints (Fig. 1A). The vaccination regimens included: BNT162b2 with a short-interval boost (> 4 weeks; n = 17), BNT162b2 with a long-interval boost (< 4 weeks; n = 19), and ChAdOx1-S with a long-interval boost (n = 20) (Table S1). Prior history of SARS-CoV-2 infection, evidenced by detectable anti-spike IgG antibodies at baseline, was balanced among the vaccine groups, and unless otherwise specifically stated, analyses focused on infection-naïve participants.

Both BNT162b2 and ChAdOx1-S vaccines elicited robust SARS-CoV-2 spike-specific T cell responses (Fig. S1A), as well as binding (Fig. S1B) and neutralizing (Fig. S1C) antibodies four weeks after each vaccination dose. Notably, modality-specific differences were observed: BNT162b2 induced higher antibody titers, whereas ChAdOx1-S induced higher T cell responses after the prime dose, which became equivalent to those induced by BNT162b2 after the boost (Fig. S1D).

### Interferons dominate early responses to SARS-CoV-2 vaccines

To profile the earliest vaccine-specific responses, we performed RNA-seq on whole blood collected before and one day after each vaccination dose.

Unsupervised principal component analysis (PCA) revealed that vaccination was the largest source of variance in the data. Specifically, the first principal component (PC1), accounting for 45.44% of the variance, distinguished pre- and post-vaccination timepoints irrespective of vaccine type (Fig. 1B). This separation was driven by interferon-stimulated genes (ISGs), such as *CXCL10* (Fig. 1C; Fig. S2A-B). Vaccine modality-specific differences only accounted for 2.96% of the variance in whole blood gene expression (PC4; Fig. S2C-E).

Among SARS-CoV-2-naïve individuals, comparison of pre- and post-vaccination samples revealed 963 differentially expressed genes (DEGs; false discovery rate (FDR) < 0.05, log2 fold change > 1) following BNT162b2 boost, compared with 627 DEGs after the prime dose. In contrast, ChAdOx1-S induced more pronounced gene expression changes at prime (1,716 DEGs) compared with boost (985 DEGs) (Fig. 1D). Most vaccine-modified genes were shared between prime and boost (Fig. 1E), and the fold changes in gene expression induced by prime versus boost vaccination were strongly correlated (Fig. S2F; BNT162b2: *r*^2^ = 0.78, p < 2.2 × 10^-16^; ChAdOx1-S: *r*^2^ = 0.86, p < 2.2 × 10^-16^), with more pronounced gene expression changes at boost for BNT162b2 and at prime for ChAdOx1-S.

To explore functional differences in vaccine responses, we conducted gene set enrichment analysis (GSEA) (34). Both vaccines triggered pathways linked to innate immune cell activation, antiviral responses, and signatures associated with T cell proliferation and B cell-mediated immunity (Fig. 1F). ChAdOx1-S prime activated a broader spectrum of immune responses, with enriched pathways including hemostasis, blood coagulation, and platelet degranulation (Fig. 1F; Fig. S2G-H). All vaccine doses consistently showed enrichment of inflammatory response and cytokine signaling pathways, including those mediated by type I IFN and IFN-γ (Fig. S2G). However, normalized enrichment scores (NES) indicated that cytokine production (Fig. S2I) and cytokine response (Fig. 1G) pathways were more pronounced following ChAdOx1-S prime compared with boost. Conversely, these pathways were heightened following BNT162b2 boost compared to its prime, suggesting that the pattern and magnitude of early cytokine responses are vaccine dose specific.

To confirm patterns of transcriptional activation, we measured plasma cytokine concentrations. Both vaccines induced secretion of IFN-γ, IL-6, monocyte chemoattractant protein-1 (MCP-1), and IL-10, with inverse patterns after homologous prime-boost (Fig. 1H). Plasma IFN-γ levels peaked 24 hours after BNT162b2 vaccination (Fig. S3A) and were highest following the boost (Fig. 1H-I). Conversely, ChAdOx1-S induced higher IFN-γ after the prime dose (Fig. 1H-I), especially in females (Fig. S3B), along with significant increases in IFN-α2 (2.6-fold, p < 0.0001; Fig. 1J) and IL-18 (p < 0.001; Fig. S3C). Although BNT162b2 did not induce detectable IFN-α2 (Fig. 1J), previous studies using more sensitive assays have detected BNT162b2-induced IFN-α2 (18). A more sensitive assay capable of detecting all IFN-α subtypes identified modest BNT162b2-induced IFN-α, albeit substantially lower than ChAdOx1-S (Fig. S3D). Plasma IFN-β was only detected after ChAdOx1-S priming (Fig. S3E); mRNA vaccines induce local IFN-β (35), with plasma induction only reported in pathological responses (22, 36). Collectively, interferons dominate early immune responses to both BNT162b2 and ChAdOx1-S vaccines, with distinct secretion patterns of IFN-γ that appear to correlate with specific rare adverse effects: myocarditis after mRNA boost and VITT following adenoviral prime. Therefore, IFN-γ and its cellular sources may critically influence the nature and severity of vaccine-induced reactogenicity.

### Universal innate-like lymphocyte activation after SARS-CoV-2 vaccination

**Figure 2:**
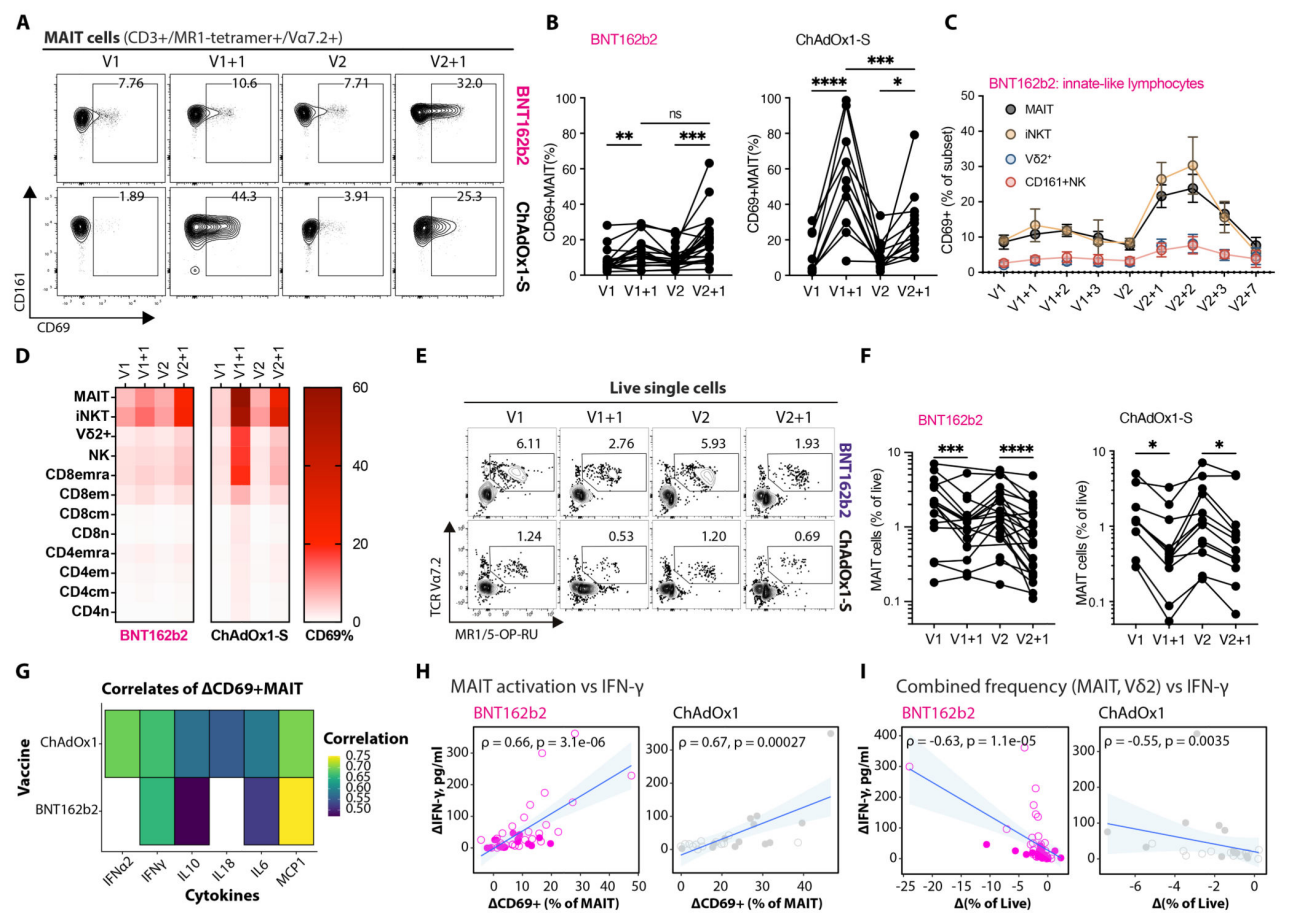
Inverse pattern of early innate-like lymphocyte responses following prime-boost with mRNA and adenoviral vector vaccines. **(A)** Flow cytometry plots showing CD69 expression on MAIT cells (MR1/5-OP-RU^+^Vα7.2^+^CD3^+^) from representative healthcare workers after receiving BNT162b2 or ChAdOx1-S. **(B)** Changes in MAIT cell CD69 expression following vaccination; statistical significance determined by mixed-effects ANOVA with Šídák’s correction for multiple comparisons. **(C)** Summary (mean ± SEM) of longitudinal CD69 expression on innate-like lymphocytes following BNT162b2 vaccination (n = 6). **(D)** Heatmap of the median CD69 expression across cell subsets at key timepoints post-vaccination with both vaccines. **(E)** Flow cytometry plots showing MAIT cell frequency (as a fraction of live cells) from representative healthcare workers before and one-day after vaccination. **(F)** Changes in MAIT cell frequency following vaccination; statistical significance determined by mixed-effects ANOVA with Šídák’s correction for multiple comparisons. **(G)** Heatmap of correlations between changes in plasma cytokine concentrations and change in CD69 expression on MAIT cells after vaccination; color indicates Spearman’s correlation coefficients for significant results (FDR < 0.05). **(H, I)** Correlation between changes in plasma IFN-γ and either (H) changes in MAIT cell CD69 expression or (I) combined frequency of MAIT cells and Vδ2^+^ γδ T cells; Spearman’s ρ and p-values are shown; symbols denote individual samples (prime, filled circles; boost, open circles; includes all donors with or without prior infection), with lines connecting measurements from the same donor. *p < 0.05; **p < 0.01; ***p < 0.001; ****p < 0.0001; ns, not significant.

CD161^+^ innate-like lymphocytes, such as MAIT cells and Vδ2^+^ γδ T cells, are highly responsive to cytokines such as type I IFNs (37, 38), exhibit robust early activation in response to ChAdOx1 vaccines, and play a role in orchestrating adaptive immunity (13, 27). We investigated their activation, measured by upregulation of CD69 expression, one day after SARS-CoV-2 vaccination (Fig. 2A). MAIT cells (CD3^+^MR1/5-OP-RU^+^Vα7.2-TCR^+^) significantly upregulated CD69 expression regardless of vaccine modality or dose (p < 0.05 to p < 0.0001; Fig. 2B), with peak activation observed after 24-48 hours (Fig. 2C). Contrasting patterns of activation were observed after the boost compared to the prime: enhanced CD69 upregulation following BNT162b2 boost, but reduced activation after ChAdOx1-S boost (Fig. 2B). This dynamic was shared across other innate and innate-like lymphocytes, including Vδ2^+^ γδ T cells, iNKT cells (Vα24-Jα18-TCR^+^), and CD161^+^ NK cells (Fig. 2D; Fig. S4A). Transient relative lymphopenia of peripheral MAIT cell frequency was observed after all vaccine doses (Fig. 2E-F) (39), contrasting with increased frequencies of intermediate (CD14^+^CD16^+^) monocytes (Fig. S4B-E) (18, 22), which may reflect cytokine-induced changes in migration or maturation.

As activated innate-like lymphocytes produce cytokines, the more abundant cell types may contribute to the early vaccine-associated inflammatory milieu; despite robust activation, iNKT cells are much rarer than MAIT cells in humans. Across both vaccine modalities, innate-like lymphocyte responses correlated with early vaccine-induced cytokines (Fig. 2G; Fig. S5A-B). Specifically, MAIT cell CD69 expression was closely linked to changes in plasma IFN-γ following vaccination with both BNT162b2 (ρ = 0.66, p < 0.0001) and ChAdOx1-S (ρ = 0.63, p = 0.00065) (Fig. 2H). A similar correlation was observed for Vδ2^+^ γδ T cell activation (Fig. S5C-D). Furthermore, across all timepoints, vaccine-induced IFN-γ inversely correlated with the change in combined frequencies of circulating MAIT cells and Vδ2^+^ γδ T cells following both BNT162b2 (r = -0.63, p < 0.0001) and ChAdOx1-S (r = -0.55, p = 0.0035) vaccination (Fig. 2I), potentially implicating IFN-γ as a broad correlate of innate-like lymphocyte functionality.

These findings suggest that the sensitivity of innate-like lymphocytes to inflammatory signals extends to vaccination (40–42), and that their effector functions may further contribute to the early cytokine response. As ChAdOx1-S induced MAIT cell activation correlates with subsequent antigen-specific T cell responses (Fig. S5E-F) (13), and early IFN-γ after BNT162b2 boost negatively correlates with the fold enhancement in antibody responses (Fig. S5G-H), early innate-like lymphocyte activation and IFN-γ production may underpin aspects of vaccine efficacy.

### Vaccine-induced cytokines signal directly to activate MAIT cells

**Figure 3:**
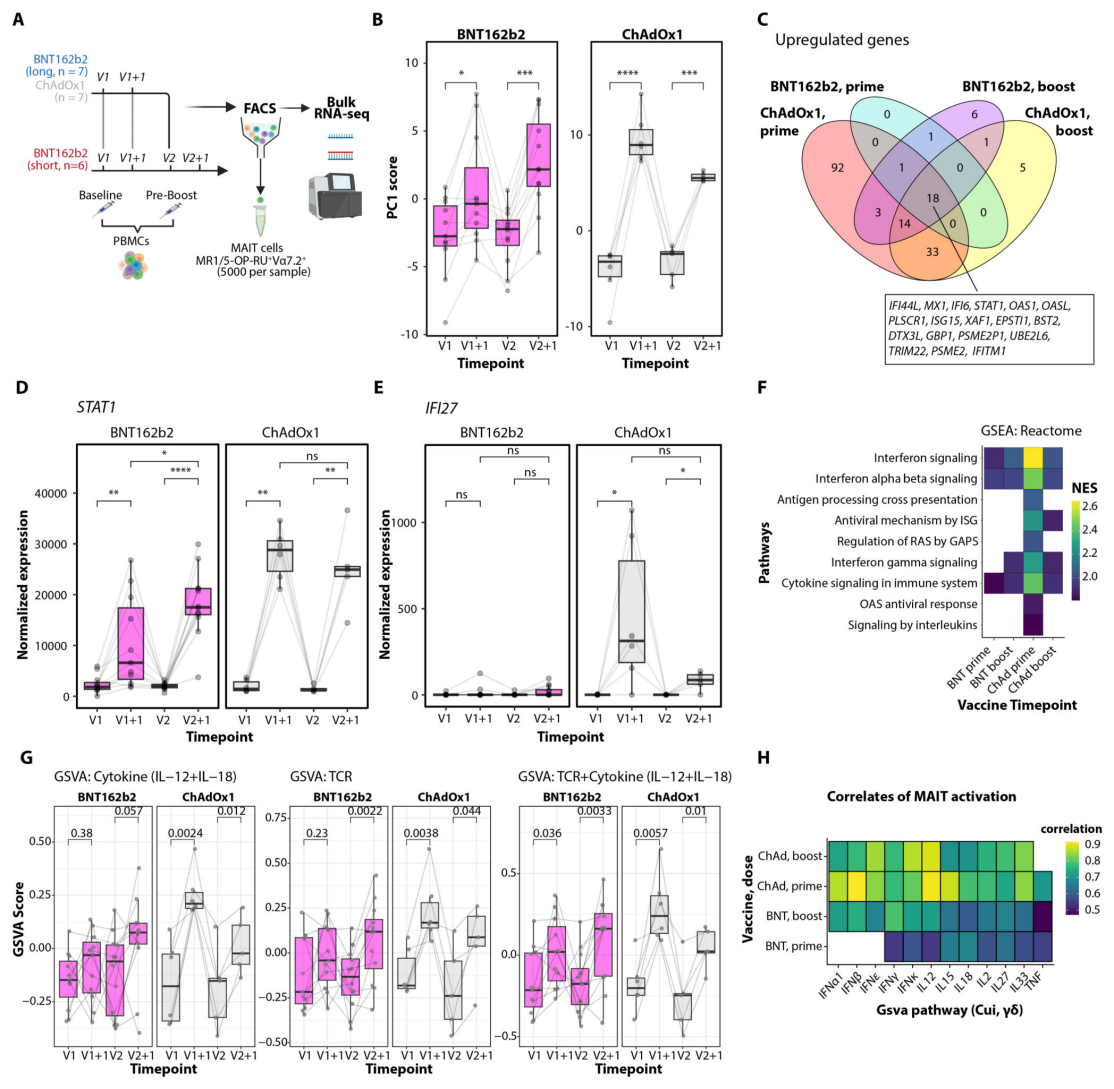
Early IFN-γ after vaccination correlates with innate-like lymphocyte activation. **(A)** Schematic for MAIT cell sorting from peripheral blood mononuclear cells (PBMCs). **(B)** Box plots of principal component 1 (PC1) scores from RNA-sequencing of MAIT cells before and after vaccination with BNT162b2 (left) or ChAdOx1-S (right) in SARS-CoV-2 naïve donors (no prior history of infection with undetectable baseline anti-S antibodies). **(C)** Venn diagrams of overlapping differentially expressed genes (DEGs; FDR < 0.05, log_2_ fold change > 0.5) in MAIT cells after prime and boost with both vaccines; shared genes across all doses are highlighted. **(D, E)** Box plots showing normalized MAIT cell expression of (D) *STAT1* and (E) *IFI27* in response to vaccination. **(F)** Gene set enrichment analysis (GSEA) normalized enriched scores (NES) for Reactome pathways enriched after each vaccine dose; only significantly enriched terms (FDR < 0.05) are shown. **(G)** Gene set variation analysis (GSVA) scores representing expression of genes from in vitro cytokine (IL-12 + IL18) and T cell receptor (TCR)-mediated activation signatures in MAIT cells (43); symbols represent individual samples with lines connecting data points from the same donor. **(H)** Correlation between changes in MAIT cell activation (CD69 expression) and changes in cytokine-specific GSVA scores of γδ T cells in vivo (44); color indicates Spearman’s correlation coefficients for significant results (FDR < 0.05). *p < 0.05; **p < 0.01; ***p < 0.001; ****p < 0.0001; ns, not significant.

To understand the vaccine-specific signals regulating MAIT cell activation and IFN-γ production, we performed RNA-seq on sorted MAIT cells (Fig. 3A). Similar to the transcriptional changes observed in whole blood, the largest variance (PC1, 26%) was due to vaccine timepoint rather than modality (Fig. 3B). Differential expression analysis confirmed global induction of ISGs after all vaccination doses, including *STAT1, ISG15, IFITM1 and MX1* (Fig. 3C), with kinetics of *STAT1* expression mirroring surface CD69 expression (Fig. 3D). ChAdOx1-S prime induced a more unique gene expression profile, upregulating *IFI27* (Fig. 3E) and *CXCL10* (Fig. S6A), along with a substantial number of downregulated genes (Fig. S6B-C). Nevertheless, pathways associated with interferon signaling were significantly enriched in response to both vaccines (FDR < 0.05; Fig. 3F), implicating the response to interferons as a key driver of early MAIT cell responses to vaccines.

To further delineate the drivers of MAIT cell transcriptional responses, we performed gene set variation analysis (GSVA) using in vitro signatures of MAIT cell activation (43), and in vivo cytokine-specific signaling signatures from murine γδ T cells (44). MAIT cells displayed enrichment for signatures of both cytokine (IL-12 + IL-18)-mediated and TCR-mediated activation (Fig. 3G). Signatures of individual cytokine signaling pathways were also enriched after vaccination (Fig. S6D) (44), and correlated with MAIT cell CD69 expression (Fig. 3H). As MAIT cell CD69 expression correlates with plasma IFN-γ (Fig. 2H), cytokine-dependent MAIT cell activation may contribute to early peripheral IFN-γ production, similar to observations after viral infection (45, 46).

### Anti-vector immunity regulates type I IFN-mediated MAIT cell responses to ChAdOx1-S

**Figure 4:**
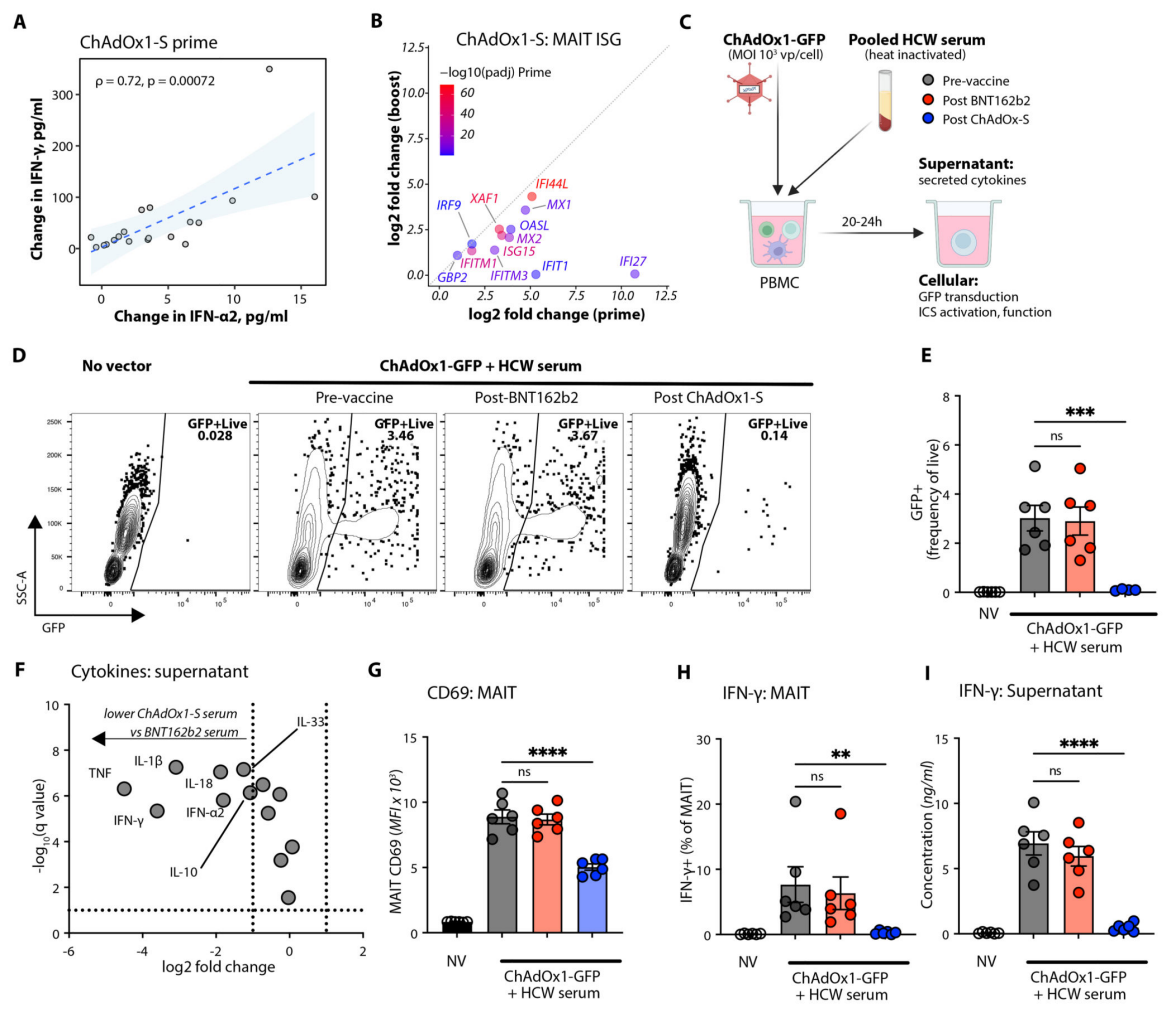
Anti-vector immunity reduces type I IFN-mediated MAIT cell activation after homologous ChAdOx1-S vaccination. **(A)** Correlation between changes in plasma IFN-α2 and IFN-γ after ChAdOx1-S prime; Spearman’s ρ and p-values are shown. **(B)** Log_2_ fold changes in MAIT cell expression of selected interferon-stimulated genes (ISGs) after ChAdOx1-S prime and boost; dotted line represents the line of identity. **(C)** Schematic of experimental design. Fresh human PBMCs (n = 6 donors; two experiments) were incubated with heat-inactivated pooled serum (10% v/v) from vaccinated SARS-CoV-2-naïve individuals and stimulated with ChAdOx1-GFP (multiplicity of infection (MOI) = 0 or 10^3^ viral particles) for 24 hours; serum sources included pre-vaccination (pooled, n = 8), post-ChAdOx1-S boost (pooled, n = 8), and post-BNT162b2 boost (pooled, n = 8). **(D, E)** (D) Representative plots and (E) summary of GFP expression as a fraction of live PBMCs. **(F)** Volcano plot of cytokines secreted after 24 hours in cell culture supernatants, comparing cells incubated with serum from ChAdOx1-S-vaccinated versus BNT162b2-vaccinated individuals; statistical significance determined using multiple paired t-tests of log-normalized values with Benjamini, Krieger, and Yekutieli correction for multiple comparisons. **(G, H)** MAIT cell (G) CD69 expression and (H) IFN-γ expression. **(I)** Concentration of IFN-γ in cell culture supernatants 24 hours after stimulation with ChAdOx1-GFP; statistical significance determined using repeated measures one-way ANOVA. Symbols represent individual samples; mean ± SEM is shown (E, G-I). *p < 0.05; **p < 0.01; ***p < 0.001; ****p < 0.0001; ns, not significant.

We next sought to understand mechanisms regulating MAIT cell responses and IFN-γ production, focusing first on ChAdOx1-S. Vaccine-induced IFN-γ strongly correlated with IFN-α (r = 0.72, p < 0.001; Fig. 4A), and MAIT cell ISG expression was less pronounced following ChAdOx1-S boost compared to prime (Fig. 4B). Given that anti-vector antibodies can reduce whole blood type I IFN signaling after homologous Ad26.COV2.S boost (30), we hypothesized that they might also regulate ChAdOx1-S-dependent MAIT cell activation after homologous boost.

To test whether anti-ChAdOx1 antibodies could modulate innate-like lymphocyte responses after ChAdOx1-S boost, we measured in vitro PBMC activation and cytokine production in response to ChAdOx1-GFP after pre-treatment with serum from individuals previously vaccinated with either BNT162b2 or ChAdOx1-S (Fig. 4C). Only pooled serum from ChAdOx1-S-vaccinated donors reduced GFP transduction in PBMCs, with no impact of anti-spike serum from BNT162b2-vaccinated individuals (Fig. 4D-E). In vitro, the presence of anti-ChAdOx1 antibodies concordantly diminished secretion of cytokines critical for ChAdOx1-GFP induced MAIT cell activation (Fig. 4F), with reductions in MAIT cell CD69 expression (Fig. 4G) and IFN-γ production (Fig. 4H), as well as total PBMC IFN-γ secretion (Fig. 4I). Therefore, anti-ChAdOx1 antibodies at boost may be responsible for reduced early immune responses after ChAdOx1-S boost. As ChAdOx1-induced MAIT cell activation is critical for antigen-specific T cell responses (13), the impact of anti-vector immunity may extend beyond early inflammatory responses to affect adenoviral vector immunogenicity.

### Amplification of antigen-specific T cell responses by innate-like lymphocyte IFN-γR1 signaling

**Figure 5:**
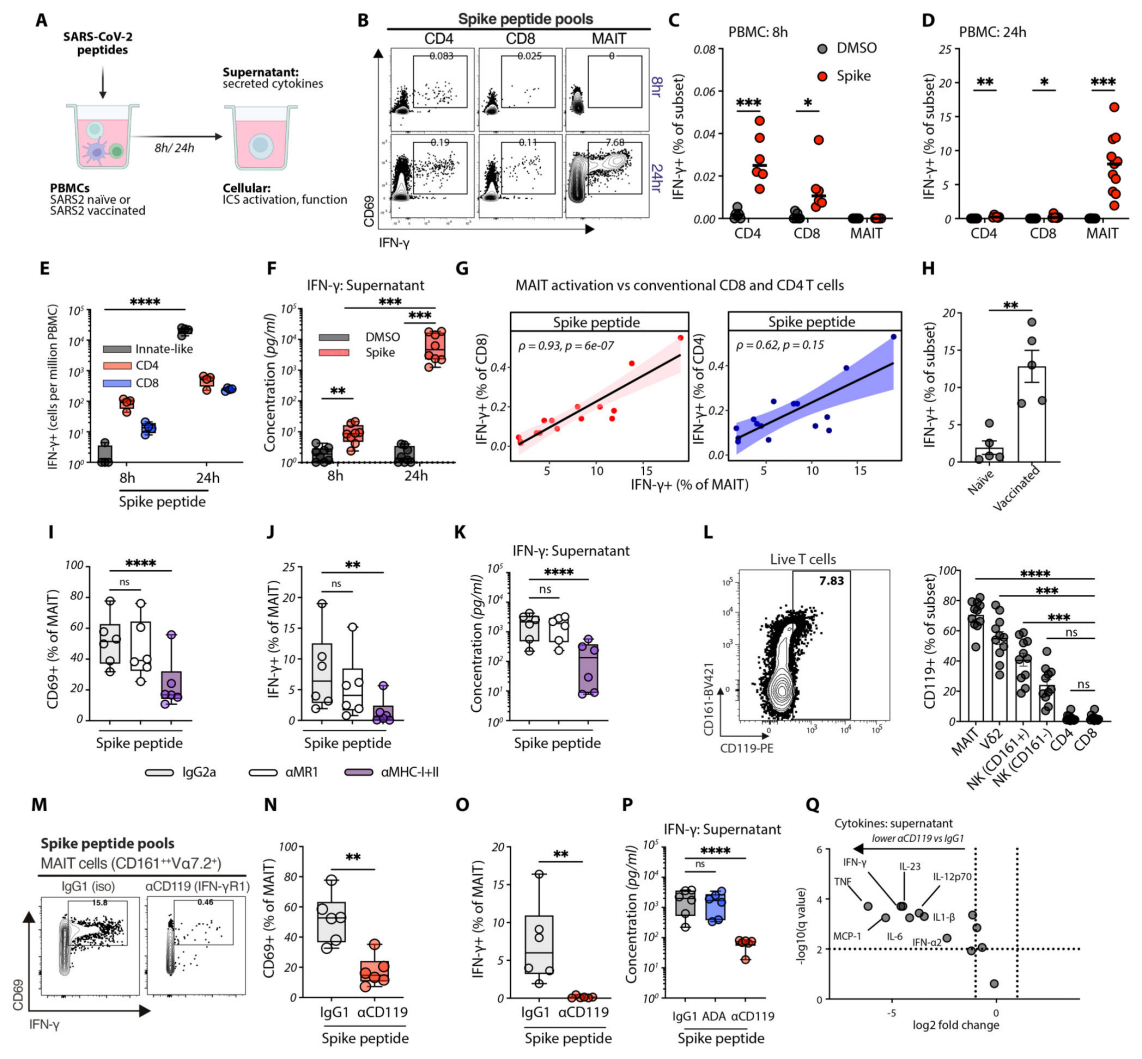
Spike-specific T cells enhance innate-like lymphocyte IFN-γ via IFN-γ receptor signaling. **(A)** Schematic of experimental design. Fresh human PBMCs from vaccinated, SARS-CoV-2-naïve individuals were stimulated with pooled S1 and S2 spike peptides (1 μg/ml total). Activation of natural killer (NK) cells (CD161^+^CD3^−^ lymphocytes), MAIT cells (CD161^++^Vα7.2^+^CD3^+^), Vδ2^+^ and Vδ2^−^ γδ T cells, and conventional CD4^+^ and CD8^+^ T cells was measured after eight or 24 hours. Inhibitors were added prior to stimulation, and cytokine concentrations were measured in supernatants after 24 hours. **(B-D)** Representative (B) flow cytometry plots and (C, D) summary data of IFN-γ expression in conventional CD4^+^ T cells, CD8^+^ T cells, and MAIT cells after (C) eight hours and (D) 24 hours of spike peptide stimulation. **(E, F)** (E) Total IFN-γ secreting cells (background subtracted cells per million live PBMCs) and (F) concentration of IFN-γ in supernatants after spike peptide stimulation of fresh PBMCs. **(G)** Correlation between IFN-γ expression in MAIT cells and conventional CD8^+^ and CD4^+^ T cells in response to spike peptide pools; Spearman’s ρ and p-values are shown. **(H)** MAIT cell IFN-γ expression following spike peptide (Peptivator) stimulation of frozen PBMCs from individuals five months post-ChAdOx1-S vaccination, compared to pre-pandemic controls. **(I-K)** PBMCs were stimulated with spike peptides with or without prior treatment with anti-MHC class I (clone W6/32) and anti-MHC class II (clone TÜ39), anti-MR1 (clone 26.2), or isotype control antibodies. (I) MAIT cell CD69 expression, (J) IFN-γ expression, and (K) IFN-γ concentration in supernatants were measured after 24 hours. **(L)** Representative flow cytometry plot and summary of CD119 (IFN-γ receptor 1) expression among human PBMCs. **(M-Q)** PBMCs were stimulated with spike peptides with or without prior treatment with blocking antibodies (isotype IgG1, anti-CD119, adalimumab (ADA)). (M) Representative flow cytometry plots of MAIT cell (N) CD69 expression and (O) IFN-γ expression measured after 24 hours. (P) IFN-γ concentration in supernatants were measured after 24 hours. (Q) Volcano plot showing log_2_ fold changes in spike peptide-induced cytokines comparing anti-CD119 pre-treatment with IgG1 control; statistical significance determined using multiple paired t-tests on log_2_-transformed values with Benjamini, Krieger, and Yekutieli correction for multiple comparisons. Statistical tests used include unpaired t-tests (C, D, H, N, O), multiple Mann-Whitney U tests (F), two-way ANOVA with Šídák’s multiple comparisons test (E, I, J), repeated measures one-way ANOVA with Šídák’s multiple comparisons test (K, P), and Friedman’s test with Dunn’s multiple comparison test (L). Box plots show median and IQR, with whiskers representing the range. *p < 0.05; **p < 0.01; ***p < 0.001; ****p < 0.0001; ns, not significant.

We next explored the mechanisms underlying enhanced MAIT cell activation and IFN-γ production after BNT162b2 boost. In mice, IFN-γ following BNT162b2 boost is dependent on conventional T cells, implicating memory T cell-derived factors (23). We hypothesized that activation of spike-specific T cells may lead to heightened IFN-γ production after mRNA boost. To test this, we performed in vitro stimulation of PBMCs from SARS-CoV-2-vaccinated individuals with spike peptide pools (Fig. 5A). As expected, only spike-specific conventional CD4^+^ and CD8^+^ T cells produced IFN-γ after a short eight-hour stimulation (Fig. 5B-C; Fig. S7A). However, after extended stimulation (24 hours), a large fraction of MAIT cells, together with other innate-like T and NK cells, also produced IFN-γ (Fig. 5B,D; Fig. S7A). Indeed, unconventional T cells and NK cells comprised the majority of IFN-γ-producing cells after 24 hours (Fig. 5E), resulting in amplified IFN-γ secretion in culture (Fig. 5F). Secondary MAIT cell IFN-γ production was specific to SARS-CoV-2 spike peptide pools (Fig. S7B-C), strongly correlated with the frequency of spike peptide-induced IFN-γ^+^ conventional T cells (r = 0.93, p = 6 × 10^-7^; Fig. 5G), and was only observed in PBMCs from SARS-CoV-2-vaccinated individuals but not in pre-pandemic controls (9.81% vs 0.84%, p = 0.004; Fig. 5H). This suggests that antigen-specific conventional T cells are necessary to initiate secondary innate-like lymphocyte activation.

To examine the mechanisms of this T cell-dependent MAIT cell activation, we first examined the role of TCR signaling on in vitro spike peptide responses using inhibitors of MR1 or classical MHC molecules. Blockade of classical MHC (MHC class I and II), but not MR1, reduced MAIT cell activation (Fig. 5I), and IFN-γ production (Fig. 5J), with similar results for other innate-like lymphocytes (Fig. S7D), conventional T cells (Fig. S7E), and total IFN-γ secretion (Fig. 5K). As spike peptide-MHC signaling, but not direct TCR signaling, was required and activation was only observed in vaccinated individuals (in the presence of spike-specific conventional T cells), we surmised that spike antigen indirectly drives bystander activation of MAIT cells through conventional T cell-derived cytokines.

We next explored potential T cell-derived cytokine signals to MAIT cells. Notably, CD161^+^ innate-like lymphocytes in PBMCs express high levels of IFN-γR1 protein (Fig. 5L) (37), suggesting that spike-specific T cell-derived IFN-γ could directly modulate MAIT cell responses after mRNA boost. In vitro, blocking IFN-γR1 signaling (anti-CD119) reduced MAIT cell activation and IFN-γ production following spike peptide stimulation (Fig. 5M-O). IFN-γR1 inhibition reduced spike peptide-derived IFN-γ from innate-like lymphocytes but not conventional T cells (Fig. S7F-G) and resulted in a 300-fold reduction in overall PBMC IFN-γ secretion after 24 hours (Fig. 5P). Inhibition of TNF signaling, another T cell-derived cytokine capable of signaling to MAIT cells (13, 47), had no impact on total spike-induced IFN-γ (Fig. 5P; Fig. S7H). Conversely, pre-treatment with supplemental IFN-γ prior to spike peptide stimulation further augmented the frequency of IFN-γ^+^ MAIT cells (Fig. S7I-K).

Functionally, IFN-γR1 blockade potently reduced secretion of a broad range of other inflammatory cytokines after spike peptide stimulation, including TNF, IL-6, and IL-1β (Fig. 5Q). IFN-γR1-driven crosstalk also likely involves monocytes with high IFN-γR1 expression (18, 23), but depleting CD161^+^ cells alone was sufficient to markedly reduce spike-induced IFN-γ and TNF (Fig. S7L). Peripheral blood MAIT cells from participants exhibited high expression of IFN-γR1 (CD119) pre-boost (Fig. S8A), with downregulation one day after BNT162b2 boost (Fig. S8B), consistent with direct IFN-γ signaling in vivo and ligand-induced internalization. Therefore, in the presence of TCR-triggered conventional T cells, IFN-γ is a key component of the early cytokine milieu that amplifies its own production – through IFN-γ production from IFN-γR1^+^ innate-like lymphocytes – linking adaptive and innate immune responses to mRNA vaccines at boost. Since MAIT cells in tonsil tissue can also produce IFN-γ in response to spike-peptide pools (Fig. S9), similar mechanisms may occur in draining lymph nodes where antigen is localized after vaccination.

### Extended dosing interval dampens early responses to BNT162b2 vaccination

**Figure 6:**
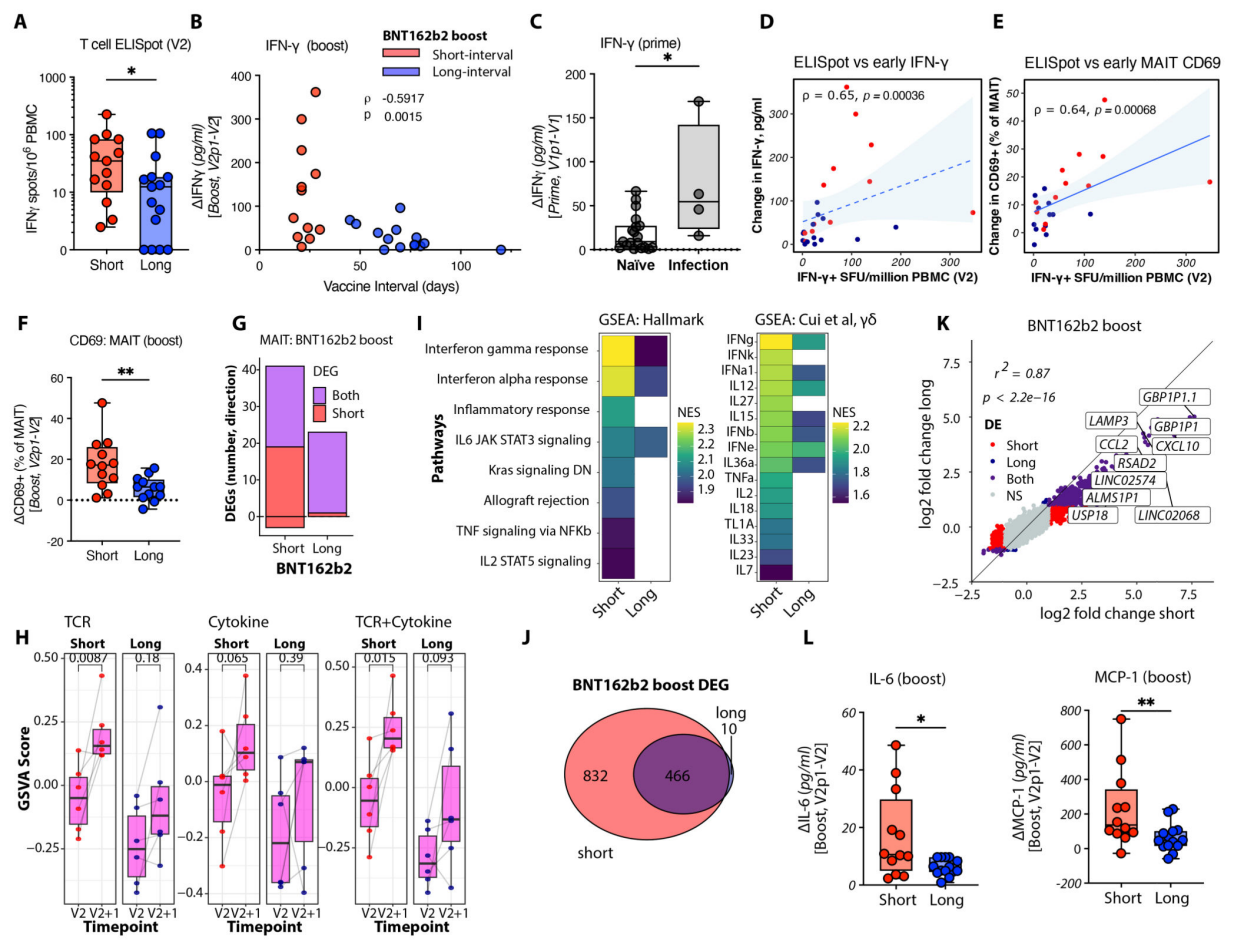
Extended BNT162b2 boosting interval reduces early innate-like lymphocyte-associated inflammatory responses. **(A)** T cell IFN-γ ELISpot responses to SARS-CoV-2 S2 peptide at the time of BNT162b2 boost (V2) in SARS-CoV-2-naïve healthcare workers boosted less than four weeks post-prime (red, short-interval boost) versus those with extended interval boosting (blue, long-interval boost); results are spot-forming units per million PBMCs. **(B)** Correlation between plasma IFN-γ induced post-boost (V2+1 minus V2) and vaccine interval in SARS-CoV-2-naïve individuals; Spearman’s ρ and p-values are shown. Samples colored by dosing interval (red, short-interval boost; blue, long-interval boost). **(C)** Plasma IFN-γ induced one day after prime in individuals with or without evidence of prior infection (positive anti-spike binding antibodies at baseline). **(D, E)** Correlations between T cell IFN-γ ELISpot (S2-specific) responses at boost and changes in (D) plasma IFN-γ and (E) MAIT cell CD69 expression after boost; Spearman’s ρ and p-values are shown. **(F)** Change in MAIT cell CD69 expression one day after BNT162b2 boost with short (≤ 4 weeks, red) or long (> 4 weeks, blue) intervals. **(G)** Number of differentially expressed genes (DEGs; FDR < 0.05, log_2_ fold change > 0.5) in MAIT cells after short-interval or long-interval BNT162b2 boost. **(H)** Gene set variation analysis (GSVA) scores representing expression of genes from in vitro cytokine-mediated (IL-12 + IL18) and T cell receptor (TCR)-mediated activation signatures in MAIT cells (43); symbols represent individual samples with lines connecting data points from the same donor. **(I)** Gene set enrichment analysis (GSEA) normalized enrichment scores (NES) for Hallmark and γδ T cell cytokine-specific gene sets (44), enriched after short-interval and long-interval boosting; only significantly enriched terms (FDR < 0.05) are shown. **(J)** Venn diagrams of overlapping DEGs at short-interval and long-interval boosts. **(K)** Log_2_ fold changes in gene expression with short- and long-interval boosts; colors indicate genes not differentially expressed (grey), or differentially expressed only with short-interval boost (red), only with long-interval boost (blue), or with both (purple); labels indicate the top 10 genes with the largest differences in fold change (filtered for genes significantly upregulated with both). Pearson’s *r*^2^ and p-values are shown. DE = differentially expressed, NS = not significant. **(L)** Change in plasma IL-6 and MCP-1 one day after boost in individuals boosted with short or long intervals. Symbols represent individual samples; box plots show median and IQR, with whiskers representing the range. Statistical significance was determined using Mann-Whitney unpaired tests (A, C, F, L). *p < 0.05; **p < 0.01; ***p < 0.001; ****p < 0.0001; ns, not significant.

We hypothesized that differences in spike-specific adaptive immunity at the time of mRNA boost may be responsible for variation in the inflammatory responses and reactogenicity observed (26). Both antibody (Fig. S10A-B) (2, 3, 48) and activation-induced marker IFN-γ^+^ T cells wane over time after BNT162b2 prime (49). In our cohort, individuals boosted with shorter intervals displayed higher SARS-CoV-2 spike-specific T cell ELISpot responses at the time of boost (Fig. 6A; Fig. S10C), and the timing of the boost inversely correlated with the magnitude of early IFN-γ at day one post-boost (Fig. 6B; Fig. S11A), suggesting that antigen-specific T cells modify vaccine-induced early cytokine production. Consistent with other reports (19, 50), individuals with prior SARS-CoV-2 infection exhibited higher serum IFN-γ one day after prime compared to SARS-CoV-2-naïve individuals (Fig. 6C).

The magnitude of pre-boost T cell ELISpot responses correlated with subsequent IFN-γ (ρ = 0.65, p = < 0.00036; Fig. 6D) and MAIT cell CD69 expression (ρ = 0.64, p = 0.00068; Fig. 6E) following BNT162b2 boost, with higher MAIT cell activation in individuals boosted with a shorter interval (short 17.8 ± 3.6%, long 5.8 ± 1.6%, p = 0.0056; Fig. 6F). Patterns for Vδ2^+^ γδ T cell CD69 expression were similar (Fig. S11B). Earlier boosting was associated with increased transcriptional activation of MAIT cells (Fig. 6G; Fig. S11C), including enhanced signatures of TCR and cytokine signaling (Fig. 6H), with the degree of vaccine-induced MAIT cell CD69 expression correlating with cell-intrinsic IFN-γ mediated signaling (Fig. S11D). Furthermore, short-interval boosting was associated with enhanced MAIT cell interferon signaling, including cell-intrinsic IFN-γ signaling (Fig. 6I). MAIT cells and other innate-like T cells and NK cells may act as intermediaries in antigen-specific enhancement of mRNA vaccine-induced inflammation by being both sensitive to and contributing to vaccine-induced IFN-γ (50).

To assess the broader implications of interval-dependent differences in early IFN-γ-dependent MAIT cell activation, we compared whole blood transcriptional responses. Short-interval boosting induced substantially greater gene expression changes (Fig. 6J), with almost two-fold higher induction of ISGs such as *MX1, IFI44, and CXCL10* (Fig. 6K; Fig. S11E), and enriched IFN signaling (Fig. S11F). Enhanced whole blood transcriptional signatures of inflammation corresponded with increased induction of plasma cytokines, including IL-6 and MCP-1 (Fig. 6L), and intermediate monocyte abundance (Fig. S11G), thus linking differences in adaptive immunity – modulated by the boosting interval – with vaccine-induced innate responses relevant to reactogenicity.

### Adaptive responses that drive innate-like responses are associated with BNT162b2-induced reactogenicity

**Figure 7:**
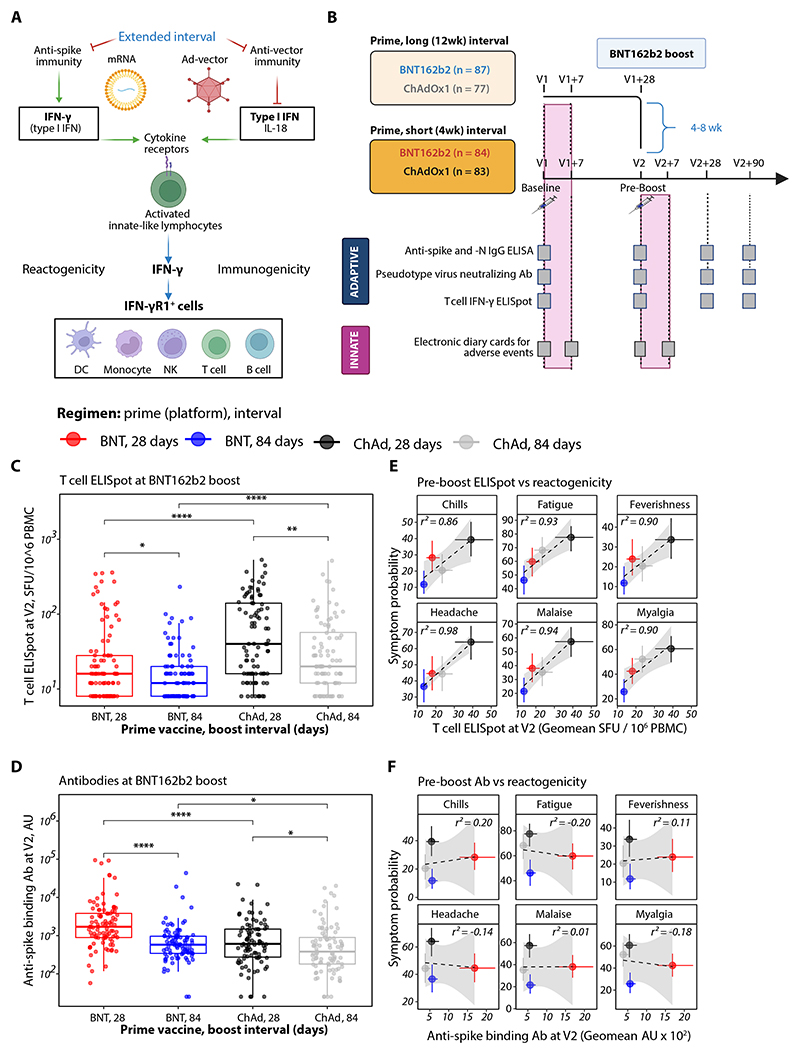
Adaptive immune correlates of BNT162b2-boost induced reactogenicity. **(A)** Model illustrating how adaptive immunity and boosting interval may regulate early innate-like lymphocyte responses to SARS-CoV-2 vaccines. **(B-E)** Reanalysis of data from the SARS-CoV-2 Com-COV trial. (B) Schematic showing the cohort receiving BNT162b2 boost; individuals received either homologous BNT162b2 prime (BNT; short-interval boost, 28 days, red; long-interval boost, 84 days, blue), or heterologous ChAdOx1-S prime (ChAd; short-interval boost, black; long-interval boost, grey), with reactogenicity documented after vaccination. (C) SARS-CoV-2 T cell IFN-γ ELISpot responses and (D) anti-spike binding antibodies at the time of BNT162b2 boost in individuals primed with either vaccine; intervals until boost were either 28 days (short) or 84 days (long). **(E, F)** Correlation between (E) T cell ELISpot responses and (F) anti-spike binding antibodies at the time of boost and the probability of systemic reactogenicity; Pearson’s *r*^2^ values are shown. Box plots show median and IQR, with whiskers representing 1.5 times the IQR. Statistical significance determined using Mann-Whitney unpaired tests (C, D). *p < 0.05; **p < 0.01; ***p < 0.001; ****p < 0.0001; ns, not significant. Schematics created in BioRender.

Systemic reactogenicity following homologous prime-boost with mRNA vaccines is reduced with extended dosing intervals (26), and correlates with the activation of NK cells and CD56^+^ innate-like T cells (20). In our model, this reactogenicity is regulated by antigen-specific T cells at the time of boost (Fig. 7A). To examine whether spike-specific T cell-mediated initiation of cytokine-responsive innate-like lymphocyte inflammatory circuits could predict systemic reactogenicity after mRNA boost, irrespective of prior vaccination modality, we analyzed data from the Com-COV trial (Fig. 7B), which evaluated short- and long-interval homologous and heterologous prime-boost strategies using BNT162b2 and ChAdOx1-S (51, 52).

Similar to our cohort, the magnitude of antigen-specific T cell (Fig. 7C) and antibody (Fig. 7D) responses waned from 4 to 12 weeks after prime. Regardless of the vaccine modality used for priming, the T cell IFN-γ ELISpot responses at the time of BNT162b2 boost directly correlated with the likelihood of subsequent severe systemic symptoms (Fig. 7E; Table S2-3). Antibody titers did not predict reactogenicity (Fig. 7F). This contrasts with homologous ChAdOx1-S boost, where delayed boosting is associated with increased reactogenicity, likely due to waning anti-vector neutralizing antibodies (30). Therefore, antigen-specific adaptive immunity at the time of vaccination crucially determines both the tolerability and efficacy of these vaccine platforms, with an emerging role for innate-like lymphocyte derived IFN-γ (Fig. S12).

## Discussion

The earliest immune responses to mRNA and Ad vector vaccines diverge due to distinct interactions between innate-like lymphocytes and adaptive immunity. These differences contribute to platform-specific patterns of immunogenicity and reactogenicity, relevant for designing effective vaccination schedules. Cytokine-driven innate-like lymphocyte effector functions are dampened by anti-vector neutralizing antibodies after Ad vector vaccination, but are enhanced by spike-specific memory T cells after mRNA boosting. Importantly, extending the boosting interval inversely regulates innate-like lymphocyte activation and their contribution to systemic inflammation and reactogenicity.

We identified a critical function of MAIT cells in orchestrating early immune responses through IFN-γ, enhancing tissue innate and adaptive responses to vaccination. In mice, early T cell-derived IFN-γ regulates innate-like responses; both TCR activation of iNKT cells (53–55) and conventional memory T cells (56) accelerate NK and iNKT cell activation and IFN-γ production, which is relevant to anamnestic responses. Similarly, 5-OP-RU activated human MAIT cells trigger NK cell activation in an IFN-γ-dependent, antigen presenting cell-independent manner (57), potentially through IFN-γ exchange at immunological synapses (58). MAIT cells are abundant at barrier sites, where crosstalk with tissue-resident memory CD8^+^ T cells could enhance IFN-γ-mediated antiviral protection (59) and mucosal vaccine efficacy (60). We also detected MAIT cell IFN-γ in ex vivo tonsil suspensions, implying similar crosstalk in lymphoid tissues. Functionally, early IFN-γ drives CD4^+^ T cell differentiation (61) and enhances CD8^+^ T cell proliferation, cytokine secretion and cytotoxicity (62–65); B cell recruitment after influenza vaccination likewise depends on IFN-γ-induced IL-6^+^ dendritic cells (66). We could not detect IFN-γ within PBMCs ex vivo, suggesting tissue-resident T and NK cells may be key sources of circulating IFN-γ (23), and that understanding how these abundant cells interact in tissues is crucial for understanding protective mucosal responses to vaccination.

MAIT cell indirect responsiveness to spike protein via IFN-γ as an activating signal may explain their unexpected presence amongst peptide-responsive PBMCs after mRNA vaccination (67, 68). Due to inter-individual, sex (69) and age-specific variation in MAIT cell frequency (70), this impacts interpretation of activation-induced marker or peptide-responsive T cell assays that infer antigen-specificity in the absence of tetramers (71). IFN-γ and IFN-γ-producing cells have been implicated in systemic reactogenicity and myocarditis after mRNA vaccines (20, 24), which are both reduced by delayed boosting (4, 26, 72). MAIT cell-derived IFN-γ could act on monocytes and other IFN-γR-expressing populations to amplify inflammation; IFN-γR blockade before BNT162b2 boost reduces innate activation without impairing adaptive responses (23), potentially offering strategies to minimize toxicity and increase tolerated dosages.

Comparing vaccine platforms, we found shared early type I IFN-dependent signaling, which was particularly exuberant after Ad vector priming. Although nucleoside modification dampens mRNA-induced type I IFN (73, 74), it remains necessary for BNT162b2-induced cellular responses (23). Short-interval BNT162b2 dosing produced memory T cells with a more proinflammatory effector phenotype (71), suggesting an adaptive consequence of elevated innate activation. Similarly, type I IFN-dependent MAIT cell effector functions are necessary for maximal ChAdOx1 nCoV-19-induced cellular responses (13). The striking IFN response after ChAdOx1 nCoV-19 prime may enhance cellular immunity at the expense of increased IFN-mediated toxicity and reactogenicity, notably in young females – the demographic with the highest incidence of severe adverse events (10). In contrast, reactogenicity rises after repeated mRNA (and protein) vaccine dosing (75) and is predicted by T cell ELISpot responses prior to BNT162b2 boost, implicating spike-reactive memory T cell IFN-γ in amplifying innate inflammation and associated systemic reactogenicity, potentially via IFN-dependent chemokines. Although symptom onset often precedes overt T cell expansion, consistent with an innate trigger, pre-existing memory T cell activation could amplify and prolong responses through IFN-γR1-dependent pathways, with systemic symptoms persisting up to a week (4). These findings align with murine data (23) and recent observations that baseline spike-specific CD4^+^ memory T cell frequencies correlate with proinflammatory cytokines after mRNA priming in infection-experienced individuals (50); pre-existing cross-reactive T cells to circulating coronaviruses may also contribute (76–79). Whether amplifying intermediary MAIT cell compartment characteristics also independently predict systemic reactogenicity, similar to young age and associated innate responses (7, 80–83), remains to be determined.

Our study was limited by a modest sample size and exclusive peripheral blood sampling from predominantly young healthy individuals. We lacked protective efficacy data, with reactogenicity analyses requiring an independent second cohort. Further work should extend to older individuals, tissue injection sites and draining lymph nodes as the sites of initiation of immune responses, along with variant-specific protective efficacy against the development of SARS-CoV-2. As we exclusively focussed on initial prime–boost regimens, additional studies to determine the impact of much longer vaccine dosing intervals and periodic annual boosting will be important.

In conclusion, IFN-driven crosstalk between memory T cells and innate-like lymphocytes such as MAIT cells governs vaccine immunogenicity and reactogenicity in a platform and interval-dependent manner. Targeted interventions modulating IFN-γ may mitigate reactogenicity while preserving robust immune protection.

## Materials and Methods

### Study design

This prospective, observational cohort study investigated mechanistic links between early innate-like and adaptive immunity following homologous prime-boost vaccination with either BNT162b2 or ChAdOx1-S using longitudinal samples from healthy adults. No formal power calculation was performed, with the sample size reflecting feasibility within the study period and aligning with comparable mechanistic vaccine studies capable of detecting large transcriptional changes. There were no interim stopping rules and participants could withdraw at any time. Eligibility criteria included healthy adults over 18 years of age working in healthcare settings, including allied healthcare professionals, laboratory staff, and medical students. Allocation to vaccine type and boost interval was dictated by the UK roll out (non-randomized), and investigators were aware of group assignments. Immune responses were characterized by flow cytometry, cytokine measurements, whole blood and sorted bulk MAIT cell RNA-seq, in addition to adaptive immune readouts (antibody titers, neutralization, T cell IFN-γ ELISpot). Further mechanistic investigation of responses to vaccines in vitro were performed using peripheral blood samples from healthy volunteers before and after either BNT162b2 (Oxford) or ChAdOx1-S (Dublin) vaccination. All in vitro experiments were performed at least twice using at least five donors.

### Ethics statement

This study was conducted in accordance with the Declaration of Helsinki (2008) and approved by: the Oxford GI Biobank Study Ethics Committee (REC Ref: 16/YH/0247, Yorkshire & The Humber Sheffield REC, approved on 29 July 2016, amended on 8 June 2020); St. Vincent’s Hospital Group Research Ethics Committee and Maynooth University Ethics (BSRESC-2024-38575); and South-Central Berkshire Research Ethics Committee (21/SC/0022). Written informed consent was obtained from all participants.

### Cohorts and sample collection

For longitudinal analysis of vaccine responses, 56 HCWs were recruited from Oxford University Hospitals NHS Foundation Trust and co-enrolled in the Protective Immunity from T Cells in Healthcare Workers (PITCH) study (2). Eighteen donor samples were also used in a contemporaneous study (71). Participants received two doses of either the BNT162b2 mRNA vaccine (30 μg intramuscularly in 300 μl; n = 36) or the ChAdOx1-S adenoviral vector vaccine (≥ 2.5 × 10^8^ infectious units intramuscularly in 500 μl; n = 20). Vaccinations occurred between December 2020 and May 2021. Boosting intervals were classified as “short” for BNT162b2 (< 4 weeks; median interval, 21 days; IQR, 21-24; range, 17-30; n = 17) and “long” for BNT162b2 (> 4 weeks; median interval, 70 days; IQR, 62-78; range, 42-120; n = 19) and ChAdOx1-S (median interval, 76 days; IQR, 70-79; range, 60-98; n = 20). Clinical metadata included vaccination dates, age, sex, ethnicity, and prior SARS-CoV-2 infection history, determined by PCR testing, symptom reporting, and serology for nucleocapsid and spike proteins (Table S1). The cohort had a female bias (64%), with a median age of 35 years (IQR, 30-43; range 21-65), and a median body mass index (BMI) of 23.4 kg/m^2^ (IQR, 20.3-26.0).

Peripheral blood samples were collected pre-prime (V1), one day post-prime (V1+1), pre-boost (V2), one day post-boost (V2+1), and at additional timepoints for adaptive immunity assessments: four weeks post-prime (V1+28), eight weeks post-prime (V1+70) for the long-interval groups, and four weeks post-boost (V2+28). Due to differing dosing intervals, V1+28 corresponds to V2 for the short-interval BNT162b2 group, while V1+70 corresponds to V2 for the long-interval BNT162b2 and ChAdOx1-S groups. Fresh blood collected in EDTA-coated Vacutainer tubes (BD Biosciences) was processed within four hours for plasma and peripheral blood mononuclear cell (PBMC) separation; fresh PBMCs were used to phenotype cellular activation, while frozen PBMCs were used for spike-specific T cell IFN-γ ELISpot assays. Serum was collected in serum separator tubes (SST; BD Biosciences) to measure SARS-CoV-2 specific binding and neutralizing antibodies. Whole blood collected in Tempus tubes (Life Technologies) was mixed vigorously to homogenize blood in guanidine hydrochloride solution and promptly frozen at −20°C for subsequent RNA extraction in batches. Samples were managed using the REDCap electronic data capture system. Detailed timing of samples within groups is summarized (Table S4). RNA-sequencing (RNA-seq) was performed on samples collected in Tempus RNA tubes or from PBMCs, as appropriate.

### Isolation of peripheral blood mononuclear cells (PBMCs), plasma and serum

PBMCs were isolated from fresh EDTA blood via density gradient centrifugation over Lymphoprep (Axis-Shield). For in vitro experiments, blood was diluted 1:1 with phosphate-buffered saline (PBS; Sigma-Aldrich) before layering over Lymphoprep; for longitudinal samples, undiluted blood was used to collect plasma. After centrifugation (973 *× g*, 30 min, 20°C, without deceleration), the PBMC layer was collected, washed with R10 medium (RPMI-1640 (Sigma-Aldrich) supplemented with 10% fetal bovine serum (FBS; Sigma-Aldrich) and 1% penicillin-streptomycin (Thermo Fisher Scientific)), followed by red blood cell lysis with ammonium-chloride-potassium (ACK) solution (BioLegend) for two minutes at room temperature. Cells were washed twice with R10 medium and either used immediately or cryopreserved in liquid nitrogen (90% FBS, 10% dimethyl sulfoxide (DMSO; Sigma-Aldrich)). Thawed PBMCs were rapidly warmed in a 37°C water bath and washed in R10 medium containing Benzonase (2 μl, ≥ 25 U/μl; Sigma-Aldrich).

Plasma was collected from the supernatant after initial centrifugation, further clarified by centrifugation (1000 *× g*, 10 min, 20°C), before storage at −80°C. Serum collected in SST was allowed to clot at room temperature for 30-60 minutes, centrifuged (1800 *× g*, 15 min, room temperature), and stored at −80°C.

### Assessment of SARS-CoV-2 specific adaptive immune responses

Immunoassays to detect SARS-CoV-2 specific binding and neutralizing antibodies, and T cell IFN-γ ELISpots, were performed as previously described (2). More details are provided in Supplementary Methods.

### Quantification of cytokines and chemokines

Cytokines and chemokines in plasma and cell culture supernatants were measured using the LEGENDplex Human Inflammation Panel (13-plex, BioLegend, #740808) following manufacturer’s instructions. Briefly, samples were thawed at room temperature, centrifuged (2000 *× g*, 10 min), then incubated with standards and capture beads in a polypropylene 96-well V-bottom plate (BioLegend) on a shaker (2 h, room temperature). Plates were washed twice, incubated with biotinylated detection antibodies (2 h, shaker, room temperature), after which streptavidin-phycoerythrin (PE) was added (30 min, shaker, room temperature). Finally, samples were washed twice, resuspended in LEGENDplex buffer and acquired on a BD LSR II cytometer (BD Biosciences) running FACSDiva (v9.0). Analysis was performed using the provided software. Samples were diluted to ensure concentrations were within the dynamic range of the assay and run in duplicate, with a maximum of one freeze-thaw cycle. The cytokines assayed were IL-1β, IFN-α2, IFN-γ, TNF, MCP-1, IL-6, IL-8, IL-10, IL-12p70, IL-17A, IL-18, IL-23 and IL-33. Some plasma samples were assessed with a dedicated type I IFN ELISA (Supplementary Methods).

### MR1 tetramer generation

Human MR1/5-OP-RU and MR1/6-FP tetramers were generated using NIH Tetramer Core Facility biotinylated monomers (https://tetramer.yerkes.emory.edu/support/protocols). Conjugation was performed to streptavidin-Brilliant Violet 421 (BioLegend, #405225).

### Pooled serum samples for in vitro experiments

Serum samples from SARS-CoV-2-naïve donors collected pre-vaccination and four weeks post-homologous prime-boost with BNT162b2 or ChAdOx1-S were pooled (n = 8 per group), heat-inactivated at 56°C for 30 minutes, cooled on ice, and used for in vitro assays.

### In vitro stimulation assays

Peripheral blood for in vitro experiments was collected in EDTA tubes from volunteers who had received two doses of BNT162b2 in Oxford. For in vitro SARS-CoV-2 spike peptide stimulation using thawed cells (Fig. 5H; Fig. S7B-C), stored samples were obtained from the Obesity Immunology Group cohort (Dublin), which included volunteers pre-pandemic (n = 5; all prior to November 2019), or four to six months post-ChAdOx1-S vaccination (n = 50) (Table S5; **Supplementary Methods**).

For in vitro stimulation with ChAdOx1-GFP, viral vectors and PBMCs were used as previously described (13) (Supplementary Methods). Fresh PBMCs (10^6^ cells/well) were incubated with ChAdOx1-GFP at an MOI of 10^3^ viral particles (vp)/ml in the presence or absence of pooled serum (10% v/v) for 20-24 hours in a 96-well U-bottom plate (Corning).

For stimulation of fresh cells with SARS-CoV-2 spike peptides, PBMCs (10^6^ cells/well) were stimulated with overlapping peptide pools (ancestral S1 (158) and S2 (157) 15-mers overlapping by 11 amino acids; 1 μg/ml each; PepMix PM-WCPV-S-1, JPT Peptide Technologies) for eight or 24 hours under similar conditions. Negative controls included DMSO at equal concentrations (< 1% v/v). For certain experiments, blocking antibodies were added at 10 μg/ml immediately prior to stimulation (Table S6). All incubations were at 37°C in 5% CO2. For measurement of intracellular cytokine production, brefeldin A (BioLegend, 5 µg/ml) and monensin (BioLegend, 2 µM) were added at 1:1000 dilution for the last four hours of stimulation, prior to sample collection for analysis.

### Flow cytometry and sorting of human MAIT cells

For immunophenotyping healthcare worker samples, fresh PBMCs (10^6^ cells/well) were stained with two separate panels for antibody staining (**Supplementary Methods;** gating strategy Fig. S13A-B). Antibody clones and concentrations are listed in Table S7. Where relevant, cells were first incubated with MR1 tetramers (1:200) and CCR7 antibodies (1:100) in 50 μl FACS buffer (PBS, 0.5% BSA (Sigma-Aldrich), 1 mM EDTA (Sigma-Aldrich)), prior to staining with viability dye (LIVE/DEAD Fixable Near-IR, Life Technologies; 1:400) for 15 min at 4°C. For surface staining, cells were incubated with antibody cocktails in 50 μl FACS buffer (30 min, 4°C), washed twice in FACS buffer, then fixed and permeabilized for 20 min at 4°C using Cytofix/Cytoperm (BD Biosciences, #554722). Cells were subsequently washed twice in Perm/Wash buffer (BD Biosciences, #554723), resuspended in FACS buffer at 4°C, and immediately analyzed on a flow cytometer.

For intracellular cytokine staining to measure cytokine production in vitro, after viability dye and surface staining, cells were fixed and permeabilized in 100 µl Cytofix/Cytoperm (20 min, 4°C), washed twice in FACS buffer, then incubated with intracellular staining antibodies resuspended in 50 µl Perm/Wash buffer (30 min, 4°C). After two additional washes, cells were stored in FACS buffer at 4°C until analyzed on a flow cytometer. Spike-specific responses were background subtracted from DMSO. Data were acquired on BD Fortessa or LSR II flow cytometers (BD Biosciences), and analyzed using FlowJo v10 (FlowJo, LLC).

For fluorescence-activated cell sorting (FACS), thawed PBMCs (2 × 10^6^/sample) were stained with MR1 tetramers (40 min, room temperature), washed twice, then incubated with surface antibodies in FACS buffer (20 min, 4°C). Dead cells were stained using SYTOX Green (Thermo Fisher Scientific; 1:6000 dilution). Viable MAIT cells (CD3^+^MR1/5-OP-RU^+^Vα7.2-TCR^+^) resuspended in PBS + 0.05% BSA were sorted on a BD FACSAria III (BD Biosciences) using an 85 μm nozzle directly into 1 ml of TRIzol (Ambion, #15596026), immediately snap frozen on dry ice, and stored at −80°C until RNA extraction.

### RNA extraction, library preparation and sequencing

RNA from sorted MAIT cell samples was extracted as previously described (84). Whole blood RNA was extracted from Tempus tubes in batches of 12-24 samples, with a mixture of vaccination groups and timepoints, using the Tempus Spin RNA Isolation Kit (Thermo Fisher Scientific). Samples were thawed, diluted in a 50 ml conical with 1× PBS, vortexed for 30 s, then centrifuged (3000 *× g*, 30 min, 4°C) to pellet RNA. Supernatants were removed and RNA pellets resuspended in RNA Purification Resuspension Solution, then transferred to purification filters for microcentrifuge RNA elution. RNA quantity and integrity was assessed using a Qubit 2.0 Fluorometer (Thermo Fisher Scientific) and Agilent TapeStation and submitted to the Oxford Genomics Centre (Centre for Human Genetics, University of Oxford) for library generation and sequencing.

RNA-seq libraries were prepared using the NEBNext Single Cell/Low Input RNA Library Prep Kit (New England Biolabs, #E6240) for MAIT cells, and the NEBNext Ultra II Directional RNA Library Prep Kit (New England Biolabs, #E7760) with Globin & rRNA Depletion (New England Biolabs, #E7750) for whole blood samples to remove globin mRNA, cytoplasmic ribosomal RNA, and mitochondrial ribosomal RNA. Libraries were sequenced on an Illumina NovaSeq 6000 platform with 150 base pair paired-end reads. Median read depths of 37 million (range 27-47) and 67 million (range 48-93) paired-end reads per sample were obtained from sorted MAIT cell and whole blood RNA-seq.

### Whole blood RNA-seq analysis

BCL files were converted to FASTQ files using Illumina bcl2fastq. FASTQ files were processed to a gene count matrix using a custom CGAT-Core (85) (v0.6.15) pipeline. Briefly, sequencing quality was assessed using FASTQC (Babraham Bioinformatics; v0.12.1), then FASTQ files trimmed to remove low quality bases and adapter sequences using Trimmomatic (86) (v0.39). Reads were aligned to the GRCh38 human genome using STAR (87) (v2.7.10b) and reads mapping to each gene quantified using featureCounts (88) (v2.0.6).

Ribosomal RNA, hemoglobin, and lowly expressed genes (edgeR) (89) (v4.2.1) were removed from the gene count matrix. Log-transformed trimmed mean of M-value (TMM)-normalized counts were generated using edgeR and corrected for donor and library preparation batch using limma (90) (v3.60.4). Principal component analysis was performed using PCAtools (v2.16.0).

Differential gene expression analysis at BNT162b2 prime (i.e. genes differentially expressed between V1 and V1+1) and boost, and ChAdOx1-S prime and boost, was performed using edgeR with the following model: ~ donor + library preparation batch + vaccine:timepoint. Differential gene expression analysis to directly compare short-interval and long-interval boosting was performed separately with the following model: ~ donor + interval:timepoint. Only donors with no evidence of prior infection and matched pre- and post-vaccine samples (i.e. V1 and V1+1 and/or V2 and V2+1) were included. DEGs were defined as those with an FDR < 0.05 and a log_2_ fold change > 1.

GSEA (34) was performed using fgsea (91) (v1.30.0) implemented in the clusterProfiler (92) R package (v4.12.2). Genes were ranked by a gene significance score (π-value = log_2_ fold change × -log_10_ FDR) (93). Gene sets from the following databases were tested: Gene Ontology (Biological Process terms) (94, 95), Reactome (96), Blood Transcription Modules (BTM_Plus) (97). Significantly enriched pathways were defined as those with FDR < 0.05.

### Sorted MAIT cell RNA-seq analysis

Analysis was performed as for whole blood RNA-seq analysis with the following modifications. Adapters were removed using FLEXBAR (v3.0) and lowly expressed genes (< 10 counts in ≥ 3 samples) removed from count matrices prior to differential gene expression analysis using DESeq2 (v1.42.1) (98). No batch effects were observed on principal component analysis; therefore differential gene expression analysis at BNT162b2 prime (i.e. genes differentially expressed between V1 and V1+1) and boost, and ChAdOx1-S prime and boost, was performed using the following model: ~ donor + vaccine:timepoint, using the likelihood-ratio test (LRT).

Differential gene expression analysis to directly compare short-interval and long-interval boosting was performed separately with the following model: ~ donor + interval:timepoint. lfcShrink was applied with the “apeglm” method to refine fold change estimates. DEGs were defined as those with an FDR < 0.05 and a log_2_ fold-change > 0.5. Gene annotation was performed using org.Hs.eg.db (v3.18.0).

The following additional gene sets were used for GSEA. Mouse genes from in vivo cytokine stimulated γδ T cells were translated to human equivalents using the Ensembl dataset in biomaRt (v2.58.2), aligning by Ensembl gene ID and excluding genes lacking corresponding human annotations (44). In vitro-stimulated human MAIT cell gene sets were obtained from (43) by taking the top 100 differentially expressed genes following TCR (MR1/5-OP-RU), cytokine (IL-12 + IL-18), or TCR and cytokine stimulation (Table S8). Human MSigDB Hallmark gene sets were filtered to include pathways related to cytokines or immune cell signaling. Genes were ranked by lfcShrink log_2_ fold change between pre- and post-vaccination timepoints. GSVA (99) was performed using the GSVA R package (v1.50.5) to determine module activity on a per sample basis.

### Analysis of Com-COV per participant data and reactogenicity

Participant-level data were reanalyzed from the Com-COV trial and described in detail (52) (Supplementary Methods).

### Data visualization

Data visualization was performed using GraphPad Prism (v9) and R packages including ComplexHeatmap (v2.20.0), enrichplot (v1.24.2), ggfortify (v0.4.17), ggplot2 (v3.5.1), ggpubr (v0.6.0), ggrepel (v0.9.5), and VennDiagram (v1.7.3).

### Quantification and statistical analysis

Sample sizes were not predetermined by statistical methods but similar to reported publications (13, 18). Randomization was not performed in the cohort selection for this observational study, with no blinding for either data collection or analysis. Statistical analyses were conducted using GraphPad Prism (v9) or R (v4.0.2-4.4.0), using all available data with no outlier analysis.

Specific statistical tests are detailed in the figure legends. Unless otherwise stated, analyses focused on infection-naïve participants. Two-tailed p-values < 0.05 were considered significant and p < 0.1 was considered a trend, with exact values reported. Non-parametric tests were generally used unless the data was normally distributed based on the Shapiro-Wilk test.

Correlations were assessed using Spearman’s rank correlation coefficient for non-parametric data and Pearson’s correlation coefficient for parametric data. Multiparameter correlation analysis was performed using the Hmisc R package (v5.1). For analysis of in vitro stimulations, SARS-CoV-2 spike protein-specific T cell responses were calculated based on background subtraction of cytokine production from unstimulated wells. Further details, including specific peptides (Table S9) in Supplementary Methods. In all statistical analyses, significance was considered as follows: *p < 0.05, **p < 0.01, ***p < 0.001, and ****p < 0.0001.

## Supplementary Material

Checklist

Supplementary excel file 1

Supplementary excel file 2

Supplementary material

## Data Availability

Further information and requests for resources and reagents should be directed to and will be fulfilled by the lead contacts, Nicholas Provine (nicholas.provine@ndm.ox.ac.uk) and Paul Klenerman (paul.klenerman@ndm.ox.ac.uk). This study did not generate new unique reagents or original software. RNA-seq data have been deposited on the Gene Expression Omnibus database (GEO) and are accessible through the accession numbers GSE304583 (whole blood), and GSE304584 (MAIT cells). Tabulated data underlying Figs. 1 to 7 and Figs. S1 to S11 are provided in data file S#. All data are available in the main text or the supplementary materials. Additional information required to reanalyze the data reported in the paper is available from the lead contacts on request.

## One sentence summary

Memory T cells drive early reactogenic responses to mRNA boosters via MAIT/innate-like T cells, which are tempered by dosing interval.
