# Supplementary material for: MAIT and other innate-like T cells integrate adaptive immune responses to modulate interval-dependent reactogenicity to mRNA vaccines
Source: Sci Immunol. Author manuscript; Available in PMC 2026 Feb 23. (PMC7618774; doi:10.1126/sciimmunol.adu3337)
Supplement: Supplementary material [file EMS212505-supplement-Supplementary_material.pdf]

Supplementary Materials for

Innate-like T cell sensing of adaptive responses impacts interval-dependent reactogenicity to mRNA vaccines

This file includes:

Supplementary Methods

Tables S1 to S9

Figs. S1 to S13

Data file S1

Supplementary Methods

Cell lines and viruses

Vero cells (ATCC CCL-81; ATCC) were cultured in high glucose Dulbecco's Modified Eagle Medium (DMEM; Sigma-Aldrich, #D5796), with supplemental 10% FBS (Sigma-Aldrich, #F9665), 100 U/ml penicillin, 100 mg/ml streptomycin (Sigma-Aldrich, #P0781), 2 mM L-Glutamine (Sigma-Aldrich, #G7513), and 2 mM GlutaMax (GIBCO, #35050061). SARS-CoV-2/human/AUS/VIC01/2020 virus stocks were generated in Vero cells at a multiplicity of infection (MOI) of 0.0001. Virus-containing supernatants were harvested at 80% cytopathic effect, centrifuged ($2200 \times g$, 10 min, 4°C), and stored at −80°C. Virus titers were determined using focus-forming assays on Vero cells, and genomic sequencing was performed on stocks (passage 5) to confirm spike protein integrity with no changes to the furin cleavage sites.

Focus reduction neutralization test (FRNT) for detection of SARS-CoV-2 neutralizing antibodies

Neutralizing antibody titers were measured using a focus reduction neutralization test (FRNT) as previously described (*96*), comparing the decrease in number of infected foci to control wells without antibodies. Serially diluted serum samples were mixed with SARS-CoV-2 (Victoria strain, Victoria strain, diluted to 4×10^3 CFU/mL, 25 µL per well resulting in 100 foci per well) and incubated at 37°C for one hour. The mixtures were added in duplicate to Vero cell monolayers in flat-bottom, 96-well, cell culture-treated microplates (Corning). After a two-hour incubation at 37°C, a 1.5% semi-solid carboxymethyl cellulose overlay (Sigma-Aldrich, #C4888) was added to limit virus diffusion. To perform a focus forming assay, infected cells were stained with human anti-nucleocapsid monoclonal antibody (mAb206, in-house, final concentration 2 µg/mL) and detected with peroxidase-conjugated goat anti-human IgG (Sigma-Aldrich, #A0170; final dilution 1:5000). Foci were visualized using TrueBlue Peroxidase Substrate (Insight Biotechnology, #5510-0030) and counted using an AID ELISpot reader and AID ELISpot software (v8.0, AID GmbH). The percentage of focus reduction and neutralization titer ($FRNT_{50}$) were calculated using probit analysis in SPSS (v26, IBM).

Immunoassays for detection of SARS-CoV-2 specific antibodies

Antibodies against SARS-CoV-2 spike (S) and nucleocapsid (N) proteins were quantified using the Abbott AdviseDx SARS-CoV-2 IgG II assays (Abbott Diagnostics). This chemiluminescent

microparticle immunoassay (CMIA) enabled both qualitative and semi-quantitative determination of IgG antibodies in human serum and plasma. Samples with anti-S IgG titers > 25,000 arbitrary units (AU)/ml were diluted, and titers < 50 AU/ml were categorized as "no response", as per manufacturer's instructions. For anti-N IgG, the manufacturer limit of detection was ≥ 1.40 AU, with levels between 0.50-1.39 AU designated as "equivocal".

T cell IFN-γ ELISpot for detection of SARS-CoV-2 specific T cells

IFN-γ ELISpot assays were performed on cryopreserved PBMCs using the Human IFN-γ ELISpot Basic kit (Mabtech, #3420-2A) as described previously (*2*) (http://www.pitch-study.org). Briefly, PBMCs were thawed, rested overnight, and plated at 2.5×10^5 cells per well in MultiScreen-IP filter plates (Millipore, #MAIPS4510) pre-coated with the ELISpot Basic anti-IFN-γ capture antibody (clone 1-D1K), and blocked with R10. Cells were stimulated with overlapping peptide pools spanning SARS-CoV-2 ancestral spike (S1 and S2), membrane (M), or nucleocapsid (N) proteins (18-mers overlapping by 11 amino acids; Mimotopes; 2 µg/ml; **Table S9**) for 16-18 hours at 37°C in a humidified incubator with 5% CO_2. Positive controls included peptide pools of cytomegalovirus, Epstein-Barr virus, and influenza (ProMix CEF; Proimmune, PX-CEF; 2 µg/ml), phytohemagglutinin-L (PHA; Sigma-Aldrich, #11249738001; 10 µg/ml), or concanavalin A (ConA; Sigma-Aldrich, #C5275; 5 µg/ml). Negative controls used DMSO at equivalent concentrations. Plates were washed and developed using biotinylated detection antibody (ELISpot Basic kit, clone 7-B6-1, 1 µg/ml), streptavidin-alkaline phosphatase, and NBT/BCIP Substrate Solution (Thermo Fisher Scientific, #34042), then rinsed with tap water before air drying. Spots were counted using an AID Classic ELISpot reader (v8.0, Autoimmune Diagnostika GmbH, Germany), and results were expressed as spot-forming units (SFU) per million PBMCs after subtracting background from negative controls.

Viral vectors

Replication incompetent E1/E3-deleted ChAdOx1-GFP adenoviral vectors were produced in HEK293 cells by the Jenner Institute Viral Vector Core Facility as previously described (*97*). Vectors were purified by $CsCl_2$ gradient ultracentrifugation, diluted to 1×10^{11} viral particles (vp)/ml in viral vector buffer (10 mM Tris, 7.5% sucrose, 150 mM NaCl, 0.1% Tween 80, pH 7.8; all reagents Sigma-Aldrich), and stored at −80°C. Quality control was performed in batches.

Obesity Immunology Group cohort study

Peripheral blood was collected in EDTA tubes from volunteers as part of the Obesity Immunology Group cohort study (St. Vincent's University Hospital, protocol 4.1: Metabolic and Immunological Links between Obesity and Systemic Inflammation). Samples were frozen at −80˚C and stored from volunteers either pre-pandemic (n = 5; all prior to November 2019), or four to six months after vaccination with ChAdOx1-S (n = 50). Inclusion criteria for vaccinated subjects were: ability to give informed consent, age ≥ 18 years, four to six months since second dose of ChAdOx1-S vaccine. Exclusion criteria included: known previous SARS-CoV-2 infection (confirmed by PCR or antigen test), treatment with certain classes of immunosuppressants (anti-TNF agents or IL-1, IL-2, IL-6, IL-17 inhibitors) within the preceding eight months, obesity induced by endocrine disorders, active lymphoproliferative disorder and any active illness or medication which in the opinion of the investigator precluded participation in the study.

For in vitro stimulation with SARS-CoV-2 spike-specific T cells using thawed cells, human PBMCs (10^6/well) from ChAdOx1-S vaccinated individuals (or pre-pandemic controls) were stimulated overnight with overlapping peptide pools to SARS-CoV-2 spike protein (PepTivator Prot_S and Prot_S1, 15 amino acids overlapping by 10, 0.6 nM each; Miltenyi Biotec, #130-126-700, #130-127-041). Selected experiments used SARS-CoV-2 N protein (PepTivator Prot_N; Miltenyi Biotec, #130-126-698). All incubations were at 37°C in 5% CO_2, with brefeldin A and monensin added for the final 4-6 hours.

Quantification of plasma type I IFN

For analysis of plasma IFN-β and IFN-α, the following kits were used as per the manufacturer's instructions: VeriKine-HS Human IFN-β ELISA Kit (PBL Assay Science, #41415), or VeriKine-HS Human IFN-α All-Subtype ELISA Kit (PBL Assay Science, #41115). All data were collected on a FLUOstar OPTIMA plate reader (BMG LABTECH). Samples were diluted to ensure concentrations were within the dynamic range of the assay and run in duplicate.

Analysis of responses in CD161-depleted PBMCs

To evaluate cytokine responses in the absence of innate-like lymphocytes, fresh PBMCs from healthy donors were analyzed either unfractionated or following depletion of CD161-positive cells. Depletion was performed using CD161-PE labelling in combination with the EasySep

Release Human Positive Selection Kit II (STEMCELL Technologies, #17654,) according to the manufacturer's instructions for cell depletion.

Collection and processing of tonsil tissue

Tonsils were collected from pediatric and adult patients undergoing elective tonsillectomy (January 2022-October 2023), excluding those on immunosuppressive therapy, with severe inflammation, or immunocompromising conditions (subproject 22/A003). Ethical approval was from Oxford Radcliffe Biobank (reference: 19/SC/0173). Collected tissues were placed in PBS, decontaminated at 4°C for ≥ 1 hour (PBS, 1% penicillin-streptomycin, 0.01% Normocin (Invivogen)), and mechanically dissociated through a 70 µm strainer. Cells were purified by Ficoll gradient centrifugation, washed in complete medium (RPMI-1640, 10% FBS, 1% nonessential amino acids (Thermo Fisher), 1% insulin/transferrin/selenium (Thermo Fisher), 0.01% Normocin (InvivoGen)), counted, cryopreserved in FBS with 10% DMSO, and stored at −150°C.

Analysis of Com-COV per participant data and reactogenicity

The Com-COV study (ISRCTN 69254139; protocol available online; approved by the South-Central Berkshire Research Ethics Committee, REC Ref: 21/SC/0022) was a phase 2, participant-blinded, randomized trial conducted across eight UK centers to assess the safety and immune responses of different COVID-19 vaccine combinations using the AstraZeneca (ChAd) and Pfizer-BioNTech (BNT) vaccines. Adults aged 50 and over, without prior SARS-CoV-2 infection, were assigned to one of four vaccine schedules (ChAd/ChAd, ChAd/BNT, BNT/BNT, BNT/ChAd) with either a 28 day or 84 day interval between doses. A subset of 100 participants was included in an immunology cohort for more detailed immune response analysis.

To generate data on antibody responses, serum samples were collected and analyzed at Nexelis (Laval, Canada) using ELISA to quantify SARS-CoV-2 anti-spike IgG titers, and a pseudovirus neutralization assay to determine neutralizing antibody titers (NT_{50}). Additionally, samples were analyzed at the UK Health Security Agency (Porton Down, UK) using ECLIA (Cobas, Roche) to quantify SARS-CoV-2 anti-nucleocapsid IgG.

For T cell responses, IFNγ-secreting T cells specific to SARS-CoV-2 spike protein epitopes were detected using a modified T-SPOT-Discovery assay performed within 32 hours of blood collection at Oxford Immunotec (Abingdon, UK). This assay utilized peptides spanning the

entire spike protein based on the Wuhan-Hu-1 sequence (YP_009724390.1) (15-mer peptides overlapping by 11 amino acids; proprietary peptides covering S1 and S2, with high homology peptides to endemic coronaviruses removed) and included the addition of T-Cell Xtend reagent to extend PBMC viability. T cell frequencies were reported as spot-forming cells per 250,000 PBMCs, with a lower limit of detection of one per 250,000 PBMCs, and results were multiplied by four to express frequencies per million PBMCs.

To track reactogenicity and adverse events (AEs), participants were equipped with oral thermometers, tape measures, and electronic diary cards, with paper versions available for those unable to use electronic formats. They were instructed on the self-assessment of AEs and the documentation of unsolicited AEs and symptom-relieving medication usage for seven days post-vaccination, or longer if symptoms persisted. Solicited local AEs included pain, tenderness, redness, warmth, itch, swelling, and induration, while systemic AEs included fever, chills, joint and muscle pains, fatigue, headache, malaise, nausea, vomiting, and diarrhea.

Supplementary Figures

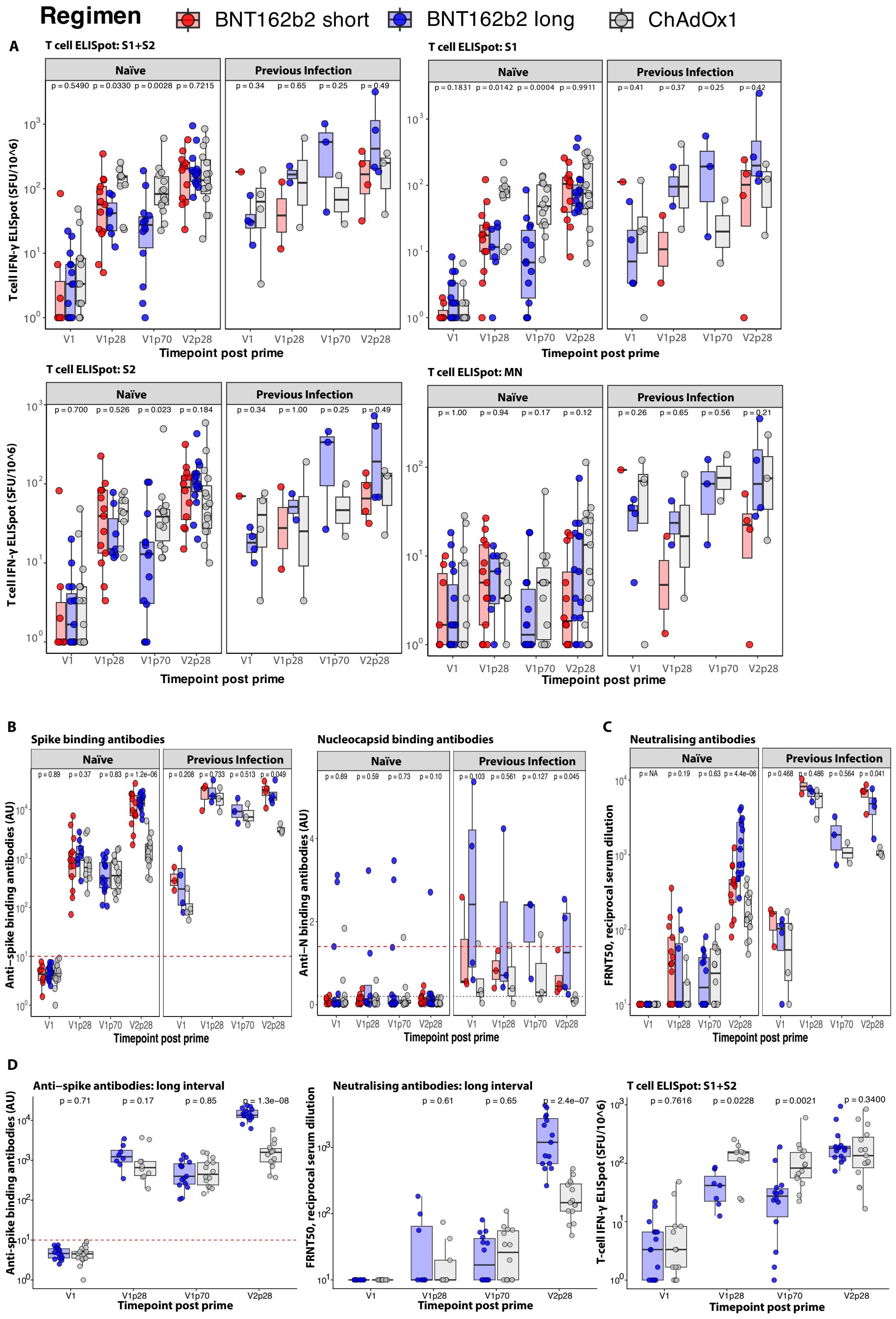

Figure S1. Adaptive immune responses to SARS-CoV-2 vaccine regimens in naïve and previously infected individuals. (A) SARS-CoV-2 spike-specific T cell IFN-γ ELISpot responses to S1 and S2 peptides or MN peptides (Victoria) using cryopreserved PBMCs; results expressed as spot-forming units per million PBMCs. **(B)** Serum SARS-CoV-2 anti-spike (S) and anti-nucleocapsid (N) IgG titers expressed as arbitrary units (AU)/ml; horizontal dotted lines indicate assay cutoffs based on pre-pandemic sera. **(C)** Neutralizing antibodies against the Victoria isolate; results presented as $FRNT_{50}$, the serum dilution required to reduce virus infectivity by 50%. **(D)** Comparison of anti-spike binding antibodies, neutralizing antibodies, and spike-specific T cell IFN-γ ELISpot responses after long-interval boosting. Statistical significance determined using Kruskal-Wallis test. Boxplots show median, first, and third quartiles, with whiskers representing the range.

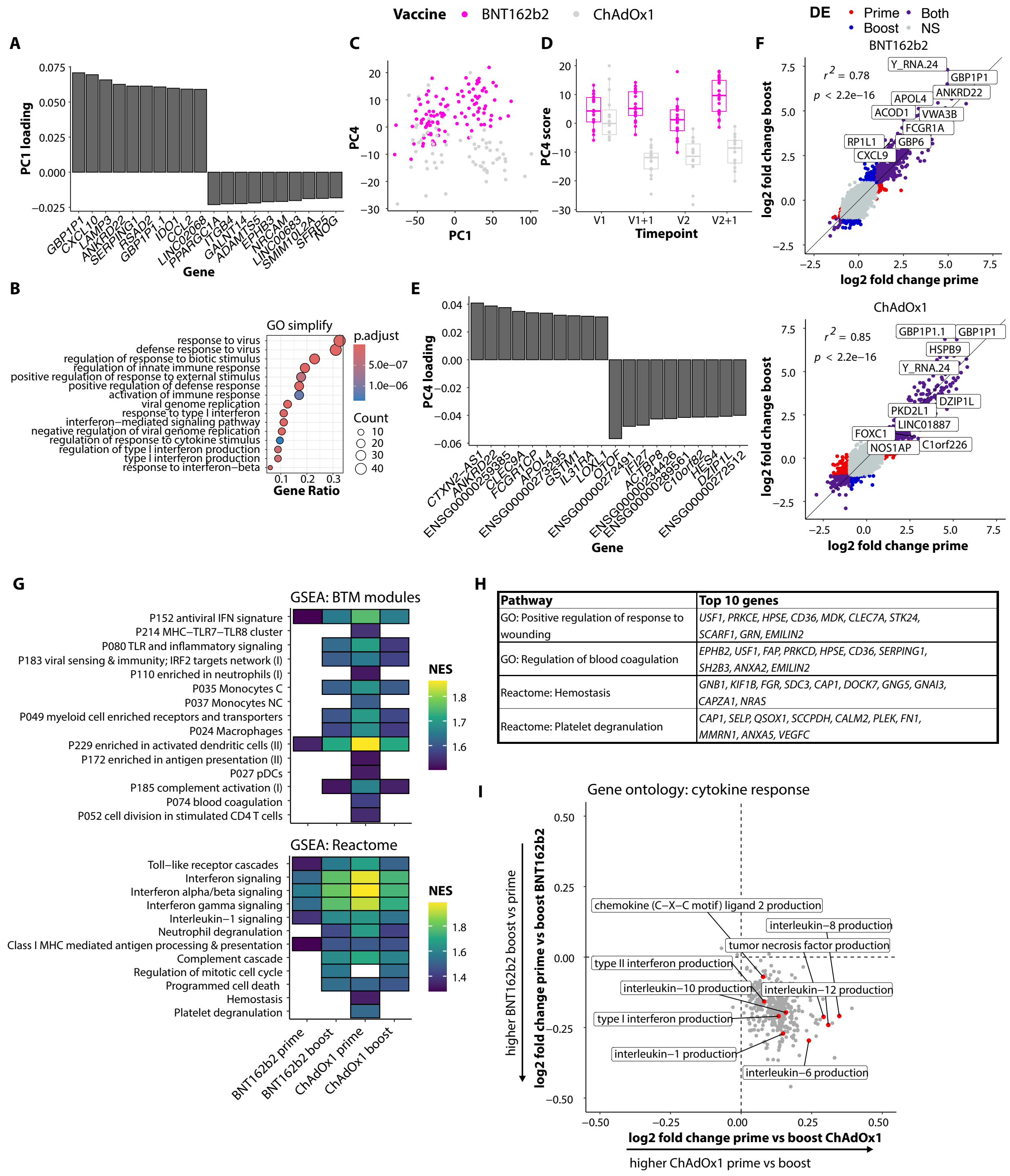

Pathway	Top 10 genes
GO: Positive regulation of response to wounding	*USF1, PRKCE, HPSE, CD36, MDK, CLEC7A, STK24, SCARF1, GRN, EMILIN2*
GO: Regulation of blood coagulation	*EPHB2, USF1, FAP, PRKCD, HPSE, CD36, SERPING1, SH2B3, ANXA2, EMILIN2*
Reactome: Hemostasis	*GNB1, KIF1B, FGR, SDC3, CAP1, DOCK7, GNG5, GNAI3, CAPZA1, NRAS*
Reactome: Platelet degranulation	*CAP1, SELP, QSOX1, SCCPDH, CALM2, PLEK, FN1, MMRN1, ANXA5, VEGFC*

Figure S2. Whole blood transcriptional response to SARS-CoV-2 vaccination. (A-D) Principal component analysis (PCA) of whole blood RNA-seq data. **(A)** Top 10 positive and negative gene loadings on principal component 1 (PC1). **(B)** Over-representation analysis on top 200 genes positively associated with PC1; top 15 Gene Ontology (GO) terms (simplified to remove redundant terms) are included with associated p-values. **(C)** Scatter plot of PC1 and PC4

scores; samples are colored by vaccine type. **(D)** Box plot of PC4 scores grouped by timepoint and vaccine type. **(E)** Top 10 positive and negative gene loadings on PC4. **(F)** Log_2 fold changes in gene expression with vaccination at prime and boost for both vaccines; colors indicate genes not differentially expressed (grey), or differentially expressed only at prime (red), only at boost (blue), or both (purple); labels indicate the 10 genes with the largest differences in fold change (filtered for genes significantly upregulated with both). Pearson's r^2 and p-values are shown. DE = differentially expressed, NS = not significant. **(G)** Gene set enrichment analysis (GSEA) normalized enrichment scores (NES) for selected Blood Transcription Modules (top) and Reactome pathways (bottom); only significantly enriched terms (FDR < 0.05) are shown. **(H)** Summary of the top 10 genes contributing to selected pathways after ChAdOx1-S prime. **(I)** Scatter plot showing log_2 fold changes in GSEA NESs for significantly enriched GO terms at prime versus boost for both vaccines; labels and red points highlight specific cytokine production pathways.

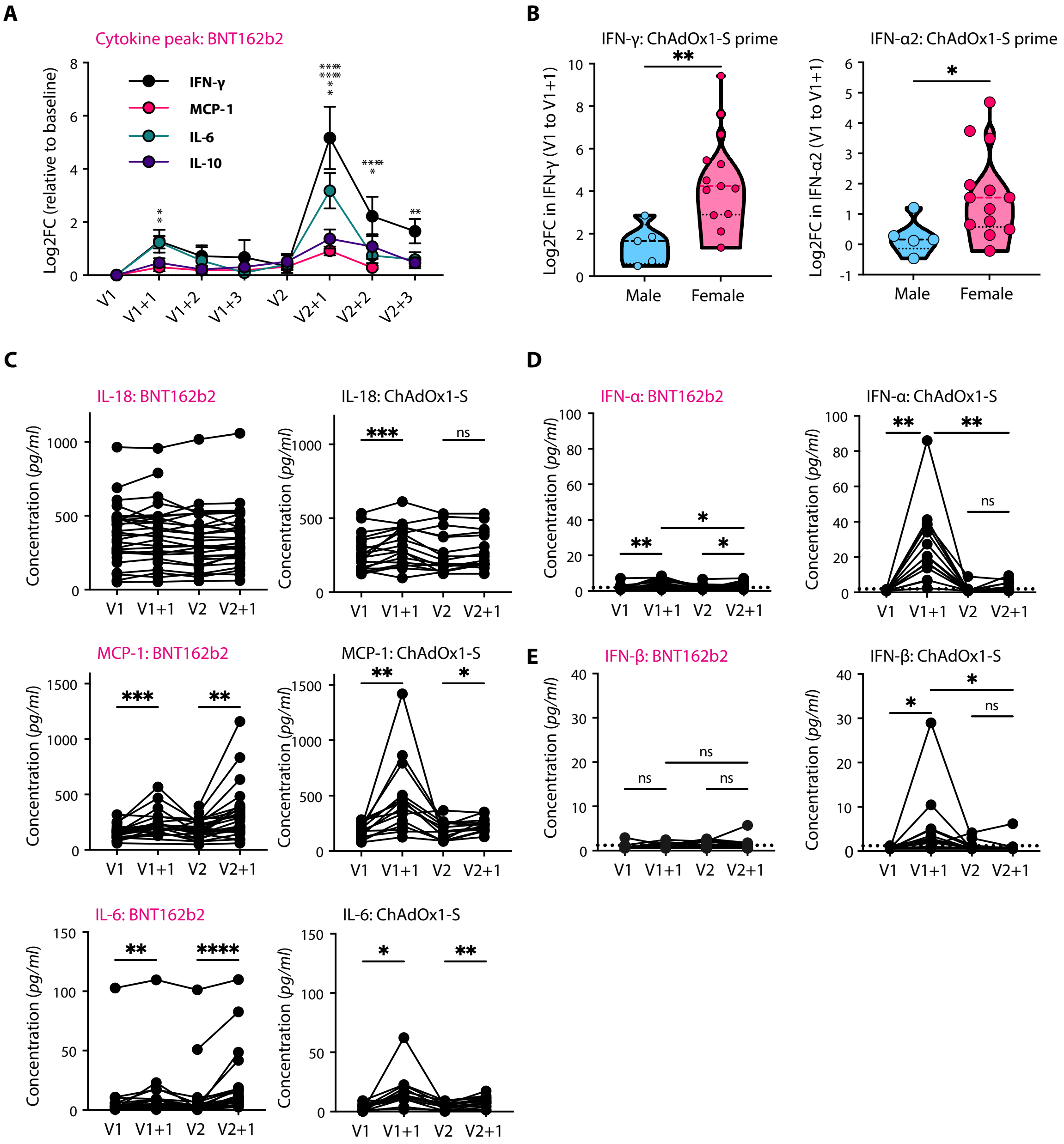

Figure S3. Plasma cytokines response to SARS-CoV-2 vaccination. (A) Longitudinal changes in plasma concentrations of IFN-γ, MCP-1, IL-6, and IL-10 after BNT162b2 vaccination; $\log_2$ fold changes relative to baseline are shown. **(B)** Changes in plasma concentrations of IFN-γ and IFN-α2 after ChAdOx1-S prime, comparing male and female volunteers; $\log_2$ fold changes relative baseline are shown. **(C)** Plasma concentrations of IL-18, MCP-1, and IL-6 from vaccinated healthcare workers at specified timepoints; symbols represent individual samples with lines connecting data points from the same donor. **(D, E)** Plasma concentrations of (D) all IFN-α

subtypes or (E) IFN-β. Statistical significance determined using (B) Mann-Whitney U test and (C) mixed-effects ANOVA with Šídák's correction for multiple comparisons. *$p < 0.05$; **$p < 0.01$; ***$p < 0.001$; ****$p < 0.0001$; ns, not significant.

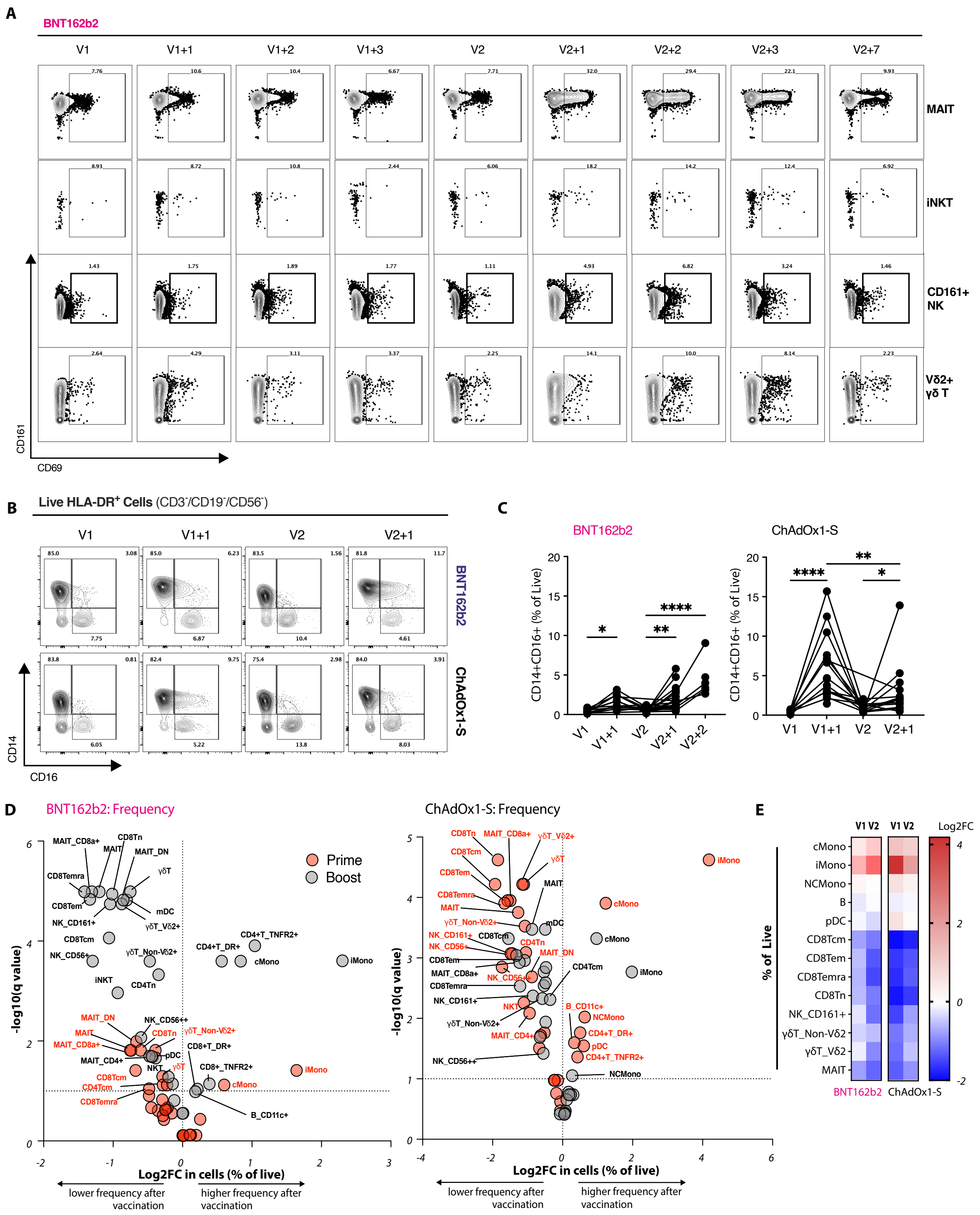

Figure S4. Differential cellular responses to BNT162b2 and ChAdOx1-S vaccines. (A) Representative flow cytometry plots from a healthcare worker receiving BNT162b2, showing

longitudinal CD69 expression on MAIT cells (CD3^{+}MR1/5-OP-RU^{+}Vα7.2-TCR^{+}), invariant natural killer T (iNKT) cells (CD3^{+}Vα24-Jα18-TCR^{+}), CD161^{+} NK cells (CD3^{-}CD161^{+} lymphocytes), and Vδ2^{+} $\gamma\delta$ T cells. **(B)** Representative flow cytometry plots gated on myeloid cells (CD3/19/56^{-}, HLA-DR^{+}) showing changes in monocyte frequency after vaccination. **(C)** Summary of changes in intermediate monocyte (CD14^{+}CD16^{+}) frequency post-vaccination; symbols represent individual samples with lines connecting data points from the same donor. **(D)** Volcano plots of $\log_2$ fold changes in cell frequency as a fraction of live PBMCs after vaccination, differentiating changes after prime (red) or boost (grey); dotted line represents FDR < 0.1. **(E)** Heatmap of median $\log_2$ fold changes in cell frequency post-prime and post-boost with both vaccines. Statistical significance determined using (C) mixed-effects ANOVA with Šídák's multiple comparisons test, and (D) multiple paired t-tests with Benjamini, Krieger, & Yekutieli correction for multiple comparisons. *$p < 0.05$; **$p < 0.01$; ***$p < 0.001$; ****$p < 0.0001$; ns, not significant. Abbreviations: pDC, plasmacytoid dendritic cell; cMono, classical monocyte (CD14^{+}CD16^{-}); iMono, intermediate monocyte (CD14^{+}CD16^{+}); ncMono, non-classical monocyte (CD14^{-}CD16^{+}); Tn, naïve T cells; Tcm, central memory T cells; Tem, effector memory T cells; Temra, RA^{+} effector memory T cells.

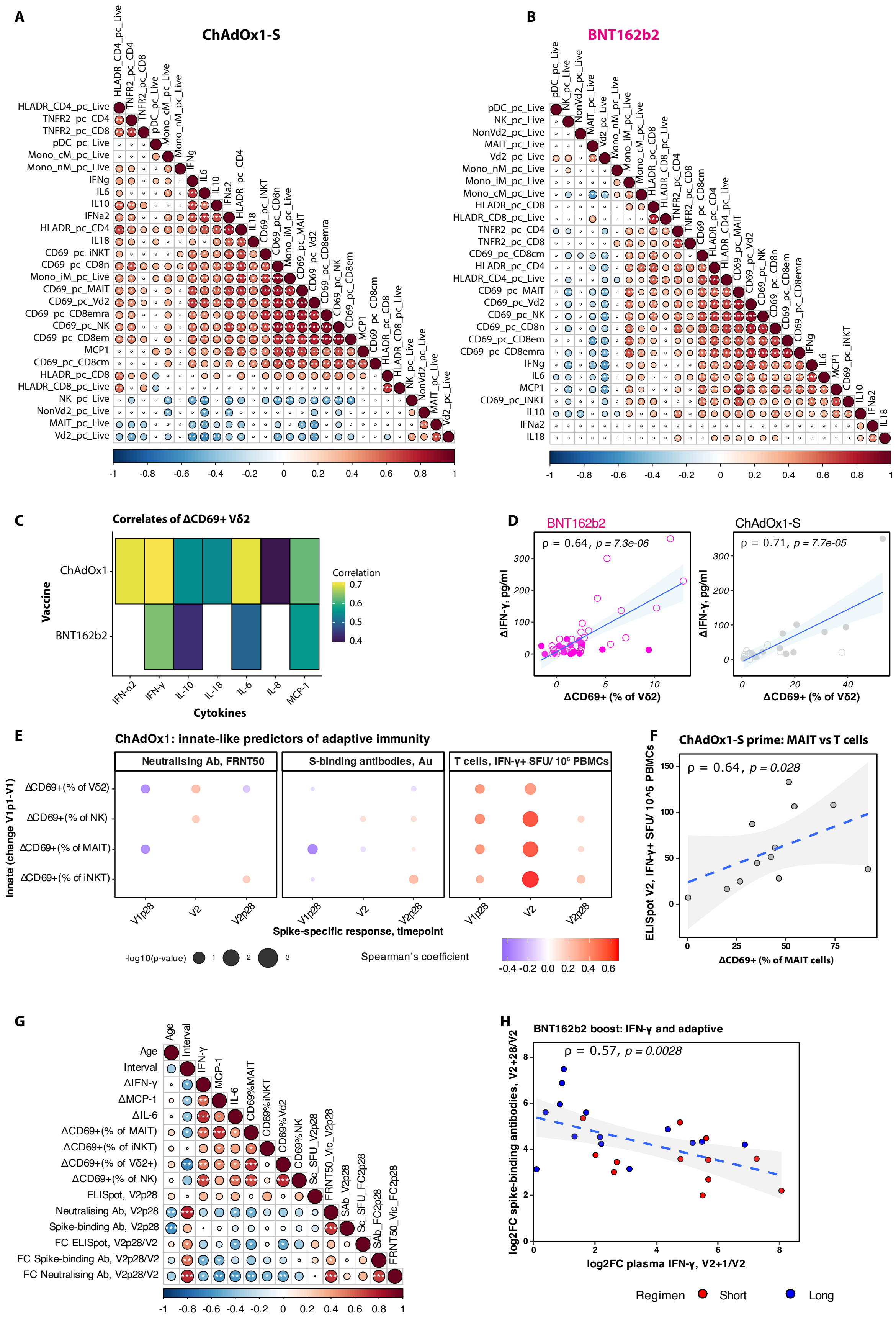
A
ChAdOx1-S
HLADR_CD4_pc_Live
TNFR2_pc_CD4
TNFR2_pc_CD8
pDC_pc_Live
Mono_cM_pc_Live
Mono_nM_pc_Live
IFNg
IL6
IL10
IFNa2
HLADR_pc_CD4
IL18
CD69_pc_iNKT
CD69_pc_CD8n
Mono_iM_pc_Live
CD69_pc_MAIT
CD69_pc_Vd2
CD69_pc_CD8emra
CD69_pc_NK
CD69_pc_CD8em
MCP1
CD69_pc_CD8cm
HLADR_pc_CD8
HLADR_CD8_pc_Live
NK_pc_Live
NonVd2_pc_Live
MAIT_pc_Live
Vd2_pc_Live
-1 -0.8 -0.6 -0.4 -0.2 0 0.2 0.4 0.6 0.8 1
B
BNT162b2
pDC_pc_Live
NK_pc_Live
NonVd2_pc_Live
MAIT_pc_Live
Vd2_pc_Live
Mono_nM_pc_Live
Mono_iM_pc_Live
Mono_cM_pc_Live
HLADR_pc_CD8
HLADR_CD8_pc_Live
TNFR2_pc_CD4
TNFR2_pc_CD8
CD69_pc_CD8cm
HLADR_pc_CD4
HLADR_CD4_pc_Live
CD69_pc_MAIT
CD69_pc_Vd2
CD69_pc_NK
CD69_pc_CD8n
CD69_pc_CD8em
CD69_pc_CD8emra
IFNg
IL6
MCP1
CD69_pc_iNKT
IL10
IFNa2
IL18
-1 -0.8 -0.6 -0.4 -0.2 0 0.2 0.4 0.6 0.8 1
C
Correlates of ΔCD69+ Vδ2
Vaccine
ChAdOx1
BNT162b2
Correlation
0.7
0.6
0.5
0.4
IFN-α2
IFN-γ
IL-10
IL-18
IL-6
IL-8
MCP-1
Cytokines
D
BNT162b2
ρ = 0.64, p = 7.3e-06
ΔIFN-γ, pg/ml
ΔCD69+ (% of Vδ2)
ChAdOx1-S
ρ = 0.71, p = 7.7e-05
ΔIFN-γ, pg/ml
ΔCD69+ (% of Vδ2)
E
ChAdOx1: innate-like predictors of adaptive immunity
Neutralising Ab, FRNT50
S-binding antibodies, Au
T cells, IFN-γ+ SFU/ 10⁶ PBMCs
Innate (change V1p1-V1)
ΔCD69+(% of Vδ2)
ΔCD69+(% of NK)
ΔCD69+(% of MAIT)
ΔCD69+(% of iNKT)
V1p28
V2
V2p28
Spike-specific response, timepoint
-log10(p-value) 1 2 3
Spearman's coefficient
-0.4 -0.2 0.0 0.2 0.4 0.6
F
ChAdOx1-S prime: MAIT vs T cells
ρ = 0.64, p = 0.028
ELISpot V2, IFN-γ+ SFU/ 10^6 PBMCs
ΔCD69+ (% of MAIT cells)
G
Age
Interval
ΔIFN-γ
ΔMCP-1
ΔIL-6
ΔCD69+(% of MAIT)
ΔCD69+(% of iNKT)
ΔCD69+(% of Vδ2+)
ΔCD69+(% of NK)
ELISpot, V2p28
Neutralising Ab, V2p28
Spike-binding Ab, V2p28
FC ELISpot, V2p28/V2
FC Spike-binding Ab, V2p28/V2
FC Neutralising Ab, V2p28/V2
-1 -0.8 -0.6 -0.4 -0.2 0 0.2 0.4 0.6 0.8 1
H
BNT162b2 boost: IFN-γ and adaptive
ρ = 0.57, p = 0.0028
log2FC spike-binding antibodies, V2+28/V2
log2FC plasma IFN-γ, V2+1/V2
Regimen Short Long

Figure S5. Correlates of early vaccine-induced innate-like lymphocyte responses. (A, B) Multi-parametric correlation matrices showing changes in immune parameters after (A) ChAdOx1-S and (B) BNT162b2, focusing on correlates of early IFN-γ; circle size and color intensity represent Spearman's correlation coefficients, with variables ordered by hierarchical clustering; includes all donors with or without prior infection. **(C)** Heatmap of Spearman's correlations between changes in plasma cytokine concentrations and change in CD69 expression on $V\delta2^{+}$ γδ T cells after vaccination; color indicates significant Spearman's correlation coefficients (FDR < 0.05). **(D)** Spearman's correlation between changes in CD69 expression on $V\delta2^{+}$ γδ T cells and concurrent changes in plasma IFN-γ after BNT162b2 (left) or ChAdOx1-S (right) vaccination; symbols denote individual samples (prime, filled circles; boost, open circles; includes all donors with or without prior infection). **(E)** Correlation of changes in innate-like lymphocyte CD69 expression (from baseline to day one post-prime) with subsequent measures of spike-specific adaptive immunity; Spearman's correlation coefficients are represented by circle size and color intensity. **(F)** Spearman's correlation between changes in CD69 expression on MAIT cells after ChAdOx1-S prime and spike-specific T cell IFN-γ ELISpot responses induced after eight weeks. **(G)** Multi-parametric matrix correlation plot for BNT162b2 boost, showing changes in plasma cytokines (IFN-γ, IL-6, MCP-1) and innate-like lymphocyte CD69 expression immediately prior and one day post-boost, focusing on subsequent measures of spike-specific adaptive immunity after four weeks; FC, fold-change; Spearman's correlation coefficients are represented by circle size and color intensity. **(H)** Spearman's correlation between $\log_2$ fold change in plasma IFN-γ after BNT162b2 boost and fold changes in anti-spike binding antibodies from boost to four weeks post-boost; symbols indicate individual donors receiving short (red) or long (blue) boosting intervals.

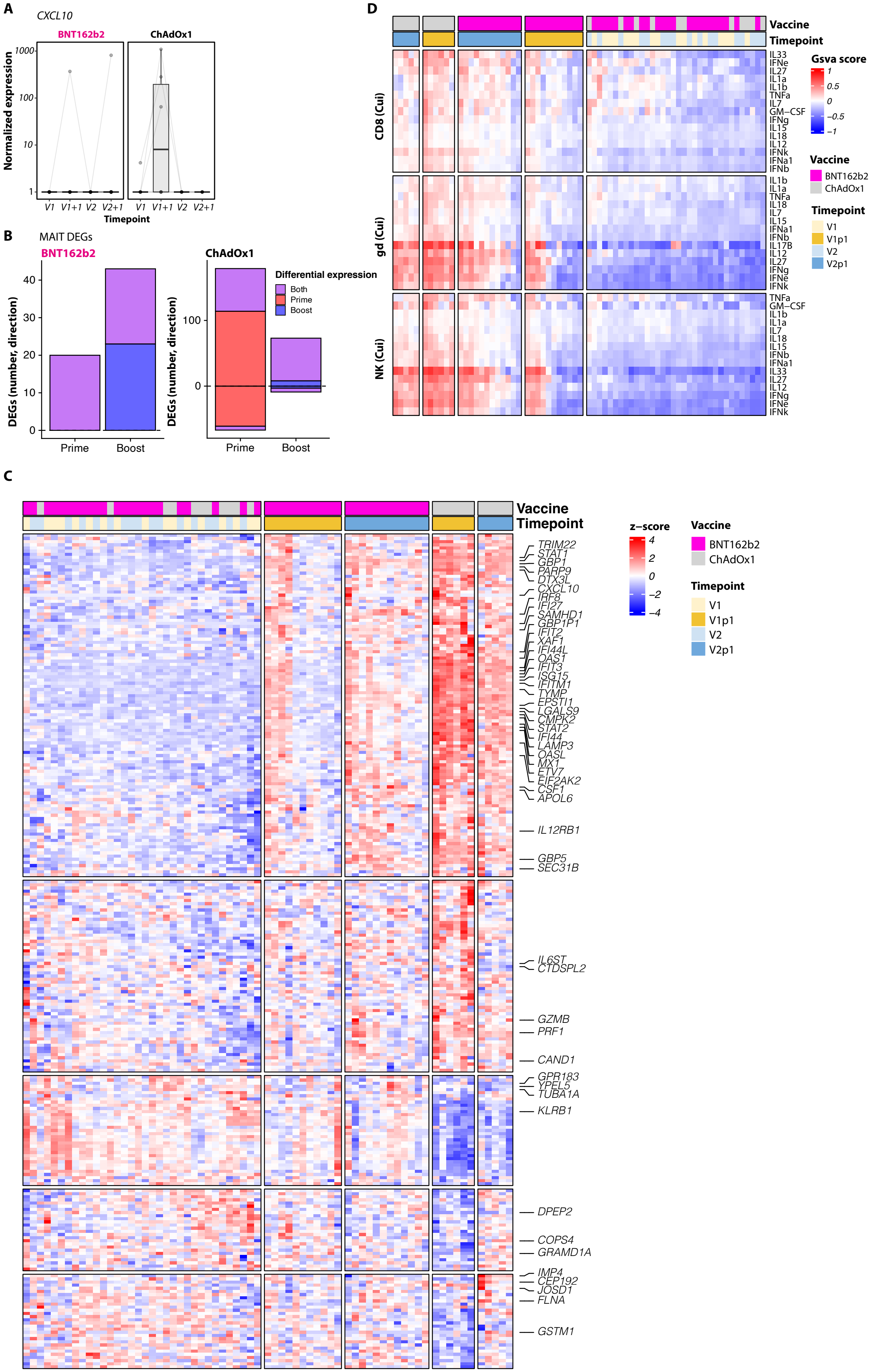
A
CXCL10
BNT162b2
ChAdOx1
Normalized expression
Timepoint
V1 V1+1 V2 V2+1
B
MAIT DEGs
BNT162b2
ChAdOx1
DEGs (number, direction)
Differential expression
Both
Prime
Boost
Prime
Boost
C
Vaccine
Timepoint
z−score
BNT162b2
ChAdOx1
V1
V1p1
V2
V2p1
TRIM22
STAT1
GBP1
PARP9
DTX3L
CXCL10
IRF8
IFI27
SAMHD1
GBP1P1
IFIT2
XAF1
IFI44L
OAS1
IFIT3
ISG15
IFITM1
TYMP
EPSTI1
LGALS9
CMPK2
STAT2
IFI44
LAMP3
OASL
MX1
ETV7
EIF2AK2
CSF1
APOL6
IL12RB1
GBP5
SEC31B
IL6ST
CTDSPL2
GZMB
PRF1
CAND1
GPR183
YPEL5
TUBA1A
KLRB1
DPEP2
COPS4
GRAMD1A
IMP4
CEP192
JOSD1
FLNA
GSTM1
D
Vaccine
Timepoint
Gsva score
CD8 (Cui)
gd (Cui)
NK (Cui)
IL33
IFNe
IL27
IL1a
IL1b
TNFa
IL7
GM−CSF
IFNg
IL15
IL18
IL12
IFNk
IFNa1
IFNb
IL17B

Figure S6. RNA-sequencing of MAIT cells after vaccination. **(A)** Normalized expression of *CXCL10* and *CSF1* in MAIT cells. **(B)** Number of differentially expressed genes (DEGs; FDR < 0.05, $\log_2$ fold change > 0.5) between pre- and post-vaccination timepoints after BNT162b2 and ChAdOx1-S prime and boost. **(C)** Heatmap showing row-scaled normalized expression of genes differentially expressed in sorted MAIT cells between pre- and post-vaccination timepoints in SARS-CoV-2 naïve individuals; selected genes are highlighted. **(D)** Gene set variation analysis (GSVA) scores representing expression of cytokine-specific genes from murine $CD8^+$ T cells, γδ T cells, $CD8^+$ T cells, and NK cells in vivo.

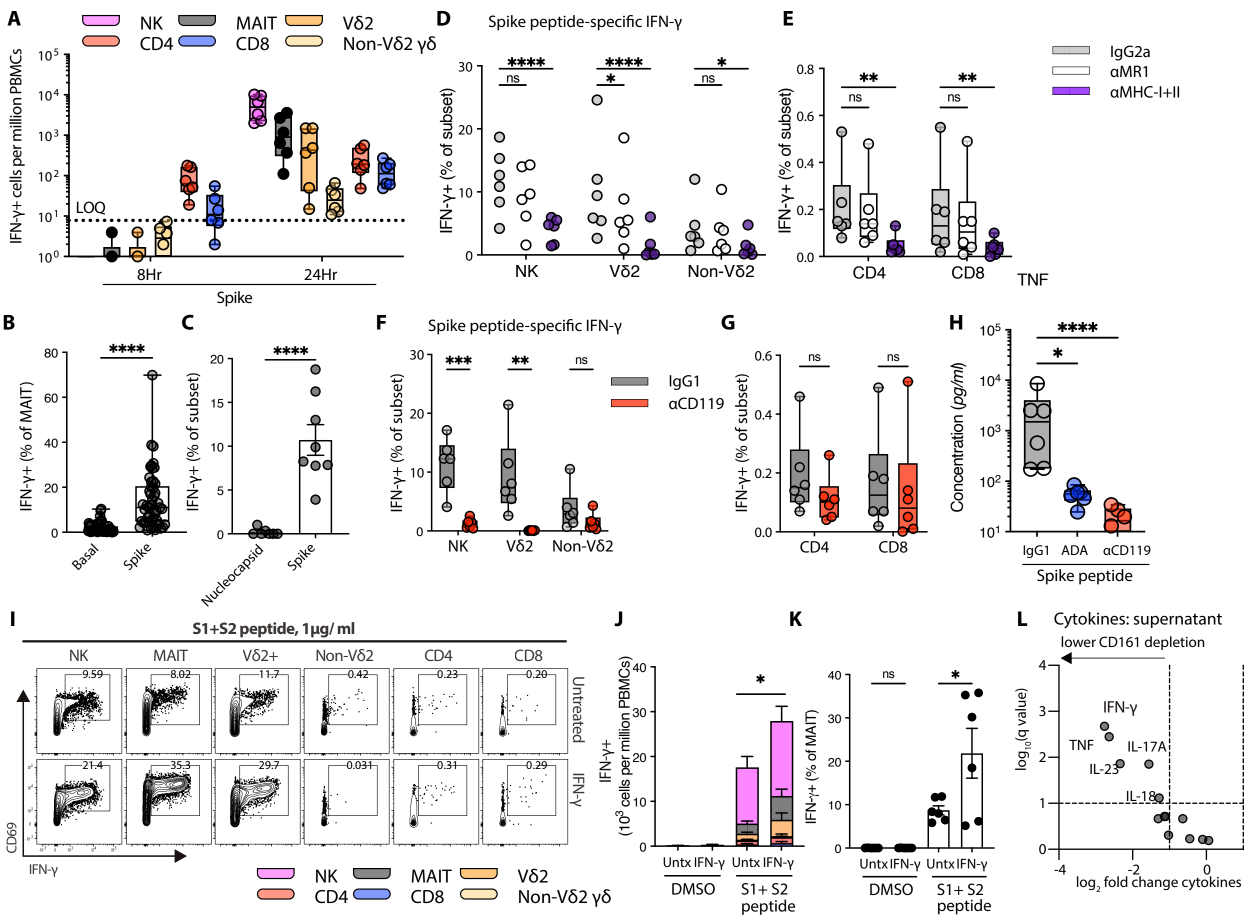

Figure S7. In vitro responses to SARS-CoV-2 spike peptide. (A) IFN-γ^+ cells (background subtracted cells per million (cpm) live PBMCs) in response to spike peptide at eight and 24 hours. **(B, C)** MAIT cell expression of IFN-γ in individuals five months post-ChAdOx1-S vaccination, following stimulation with peptide pools; comparisons include (B) control and SARS-CoV-2 spike peptide pools, or (C) SARS-CoV-2 nucleocapsid and spike-peptide pools. **(D-G)** IFN-γ expression in (D, F) selected innate-like lymphocytes ($CD161^+$ NK cells, $V\delta2^+$ and $V\delta2^-$ γδ T cells), and (E, G) conventional $CD4^+$ and $CD8^+$ T cells in response to spike peptide pools (D, E) with or without pre-treatment with 10 µg/ml of anti-MHC class I (clone W6/32) combined with anti-MHC class II (TÜ39), anti-MR1 (clone 26.2), or isotype control IgG2a antibodies, or (F, G) anti-CD119 (IFN-γ receptor 1) or isotype control IgG1 antibodies. **(H)** TNF concentration in response to spike peptide pools with or without prior treatment with anti-CD119 (red) or Adalimumab (ADA, blue). **(I)** Representative flow cytometry plots of CD69 and IFN-γ expression on NK cells, MAIT cells, $V\delta2^+$ and $V\delta2^-$ γδ T cells, and conventional $CD4^+$ and $CD8^+$ T cells in response to spike peptide pools with or without IFN-γ pre-treatment. **(J)** IFN-γ^+

cells (background subtracted cpm live PBMCs) and **(K)** fraction of IFN-γ^+ MAIT cells in response to spike peptide stimulation with or without additional IFN-γ treatment. **(L)** Volcano plot showing $\log_2$ fold changes in spike peptide-induced cytokines comparing CD161-PE depleted PBMCs with control PBMCs; statistical significance determined using multiple paired t-tests on $\log_2$-transformed values with Benjamini, Krieger, and Yekutieli correction for multiple comparisons. All donors were SARS-CoV-2-naïve prior to vaccination. Box plots show median and IQR, with whiskers representing the range. Statistical analyses performed using (B) Wilcoxon test, (C, F, G) unpaired t-tests, (D, E) two-way ANOVA with Dunnett's correction for multiple comparisons, (H, K) repeated measures one-way ANOVA with Šídák's correction for multiple comparisons. *$p < 0.05$; **$p < 0.01$; ***$p < 0.001$; ****$p < 0.0001$; ns, not significant.

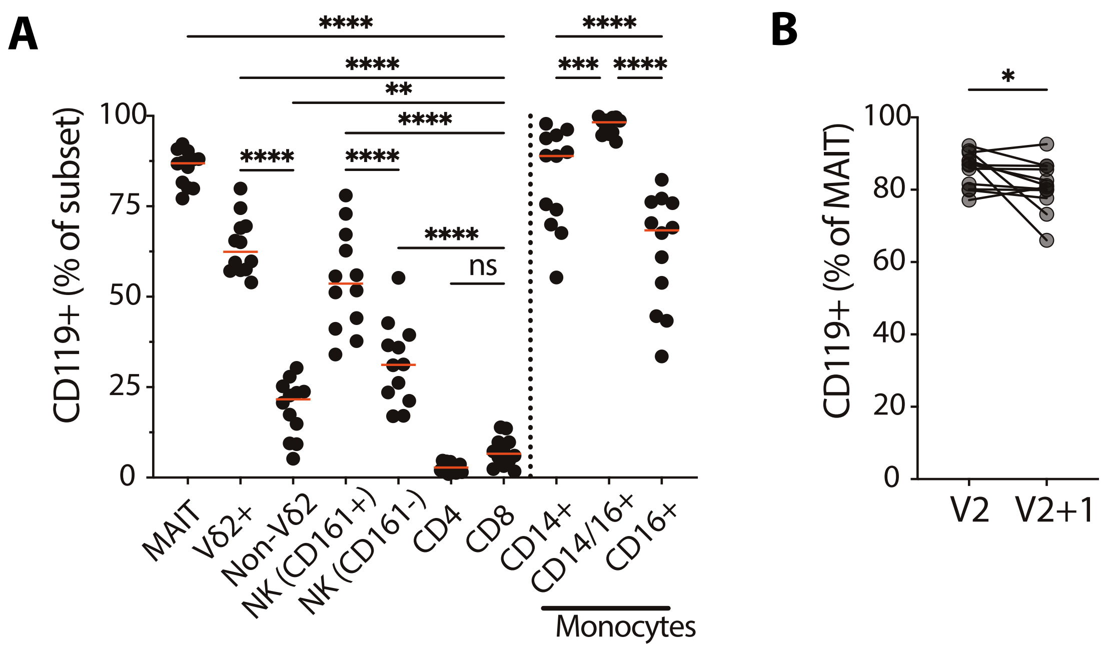

Figure S8. IFN-γR1 expression on healthcare worker samples. Summary of CD119 (IFN-γ receptor 1) expression on human PBMCs from healthcare workers receiving BNT162b2 vaccination. **(A)** Expression at pre-boost (V2) timepoint across cell subsets. **(B)** Change in CD119 expression after vaccination in MAIT cells. Statistical significance determined using (A) repeated measures one-way ANOVA, and (B) Wilcoxon test. *$p < 0.05$; **$p < 0.01$; ***$p < 0.001$; ****$p < 0.0001$; ns, not significant.

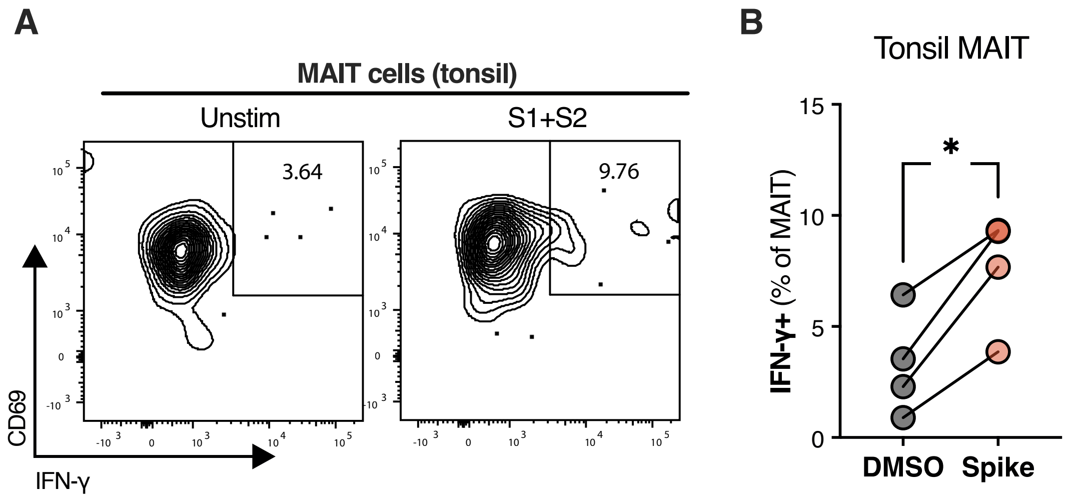

Figure S9. Tonsil-derived MAIT cells produce IFN-γ in response to spike peptide stimulation. (A, B) Frozen human tonsil tissue from SARS-CoV-2-spike antigen-exposed (vaccinated or infected) individuals was stimulated with pooled S1 and S2 spike peptides (1 μg/ml total) or DMSO control; activation of MAIT cells ($CD3^{+}CD161^{++}V\alpha7.2\text{-}TCR^{+}$) was measured after 24 hours. (A) Representative flow cytometry plots and (B) summary data (B) of IFN-γ expression in MAIT cells after 24 hours of stimulation. Statistical significance determined using paired t-test. *$p < 0.05$.

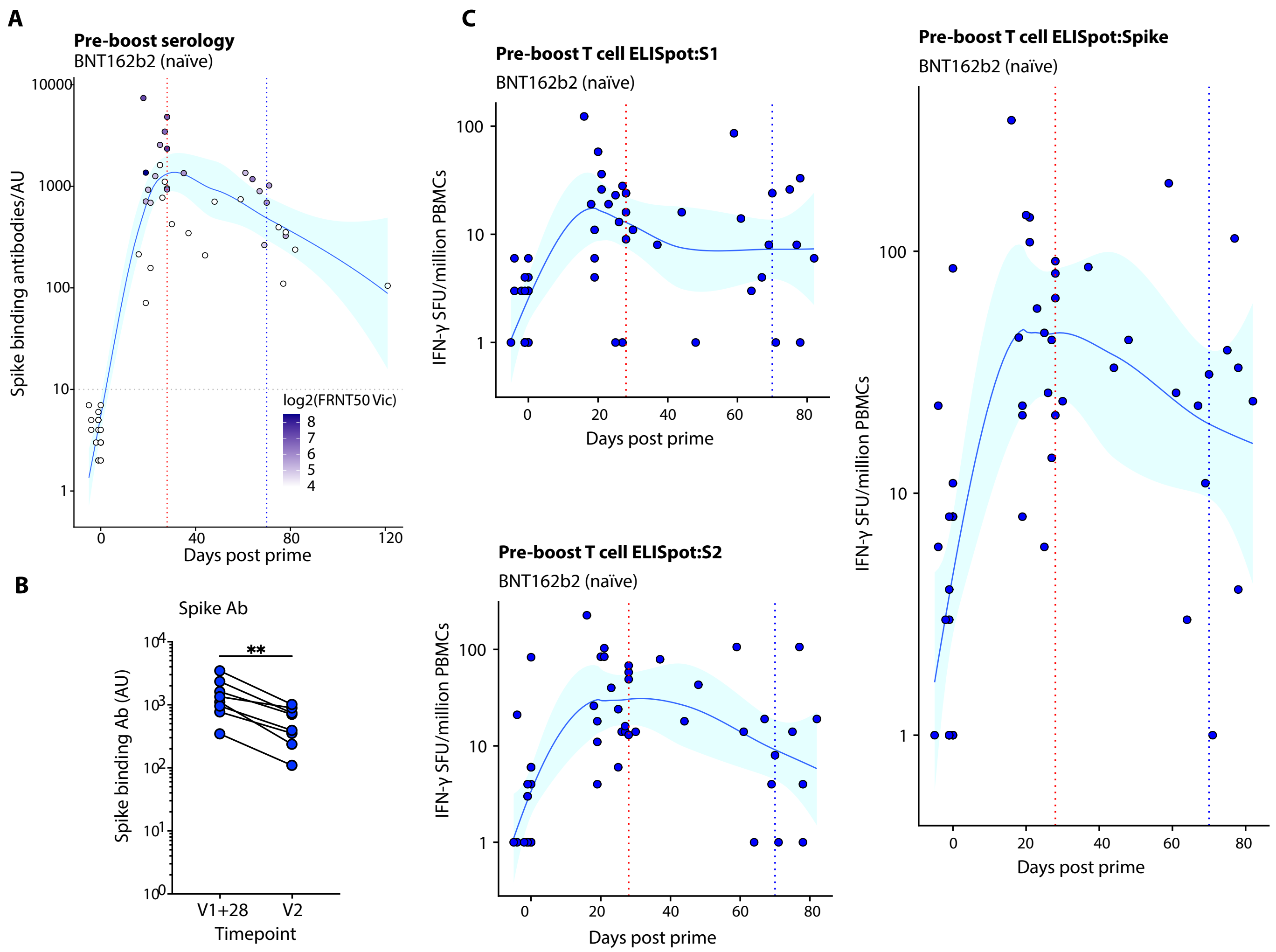

Figure S10. Comparison of spike-specific adaptive immunity at the time of boost in healthcare workers with extended boosting intervals. (A) Concentration of SARS-CoV-2 spike-binding antibodies at indicated days post-prime; dotted red and blue lines indicate average times of boost with short-interval (≤ 4 weeks) or long-interval (> 4 weeks) boosting, respectively; intensity of fill corresponds to $\log_2$ neutralizing antibody titer against live Victoria isolate of SARS-CoV-2 (open circles indicate values below the limit of detection). **(B)** SARS-CoV-2 spike-specific IgG binding antibody titers at 28 days post-prime (V1+28) and at the time of boost (V2) in donors on the long-interval regimen; lines connect samples from individual donors. **(C)** IFN-γ ELISpot responses to SARS-CoV-2 spike peptides (Victoria), individual or pooled S1 and S2 peptides (Spike, S1+S2), at specified time points after BNT162b2 prime; dotted red and blue lines indicate average time of boost on short-interval (≤ 4 weeks) or long-interval (> 4 weeks) regimens, respectively; data are spot-forming units (SFU) per million

PBMCs. Donors were SARS-CoV-2 naïve prior to vaccination. (B) Statistical significance determined using Wilcoxon signed-rank test. **$p < 0.01$.

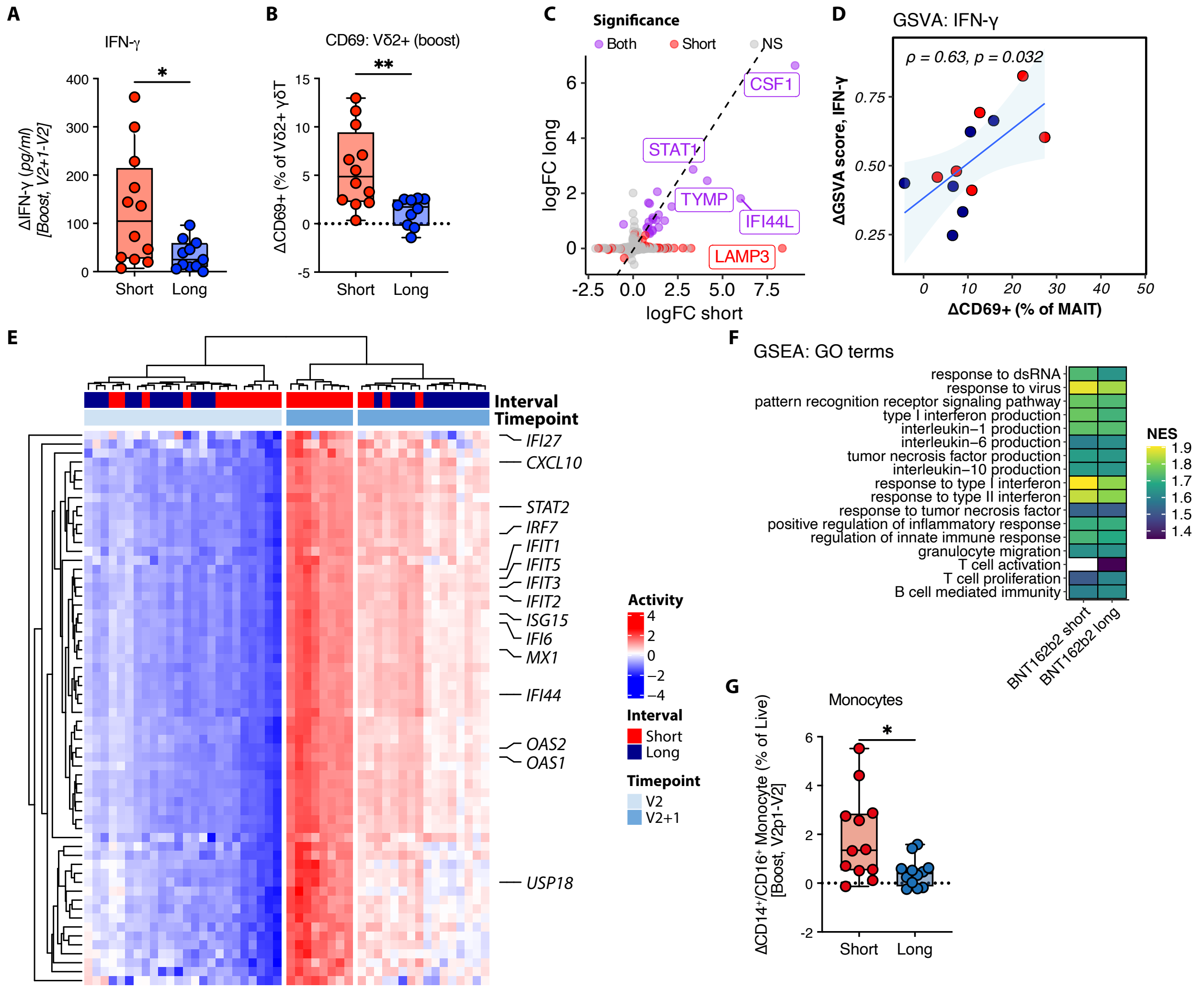

Figure S11. Higher IFN-γ and innate-like T cell activation after shorter BNT162b2 boosting intervals. (A) Change in plasma IFN-γ one day after BNT162b2 boost in individuals boosted with short (≤ 4 weeks) or long (> 4 weeks) intervals. **(B)** Change in V$\delta 2^+$ γδ T cell CD69 expression as a fraction of cells one day after BNT162b2 boost in individuals boosted with short or long intervals. **(C)** Log_2 fold changes in gene expression with short-interval and long-interval BNT162b2 vaccination; colors indicate genes not differentially expressed (grey), or differentially expressed with short interval only (red), or both (purple); labels indicate the top five genes with the largest differences in fold change (filtered for genes significantly upregulated with both). **(D)** Spearman's correlation between changes in MAIT cell CD69 expression and gene set variation analysis (GSVA) scores for IFN-γ signaling at boost. **(E)** Heatmap of protein-coding genes significantly upregulated (FDR < 0.05, log_2 fold change > 1) with short compared to long interval BNT162b2 boost; selected interferon-stimulated genes are highlighted. **(F)** Heatmap of

gene set enrichment analysis (GSEA) normalized enriched scores (NES) for selected Gene Ontology Biological Process terms; only significantly enriched terms (FDR < 0.05) are shown. **(G)** Change in intermediate ($CD14^{+}CD16^{+}$) monocyte frequency as a fraction of live cells one day after BNT162b2 boost in individuals boosted with short or long intervals. (A, B, G) Statistical significance determined using Mann-Whitney unpaired tests. *p < 0.05; **p < 0.01.

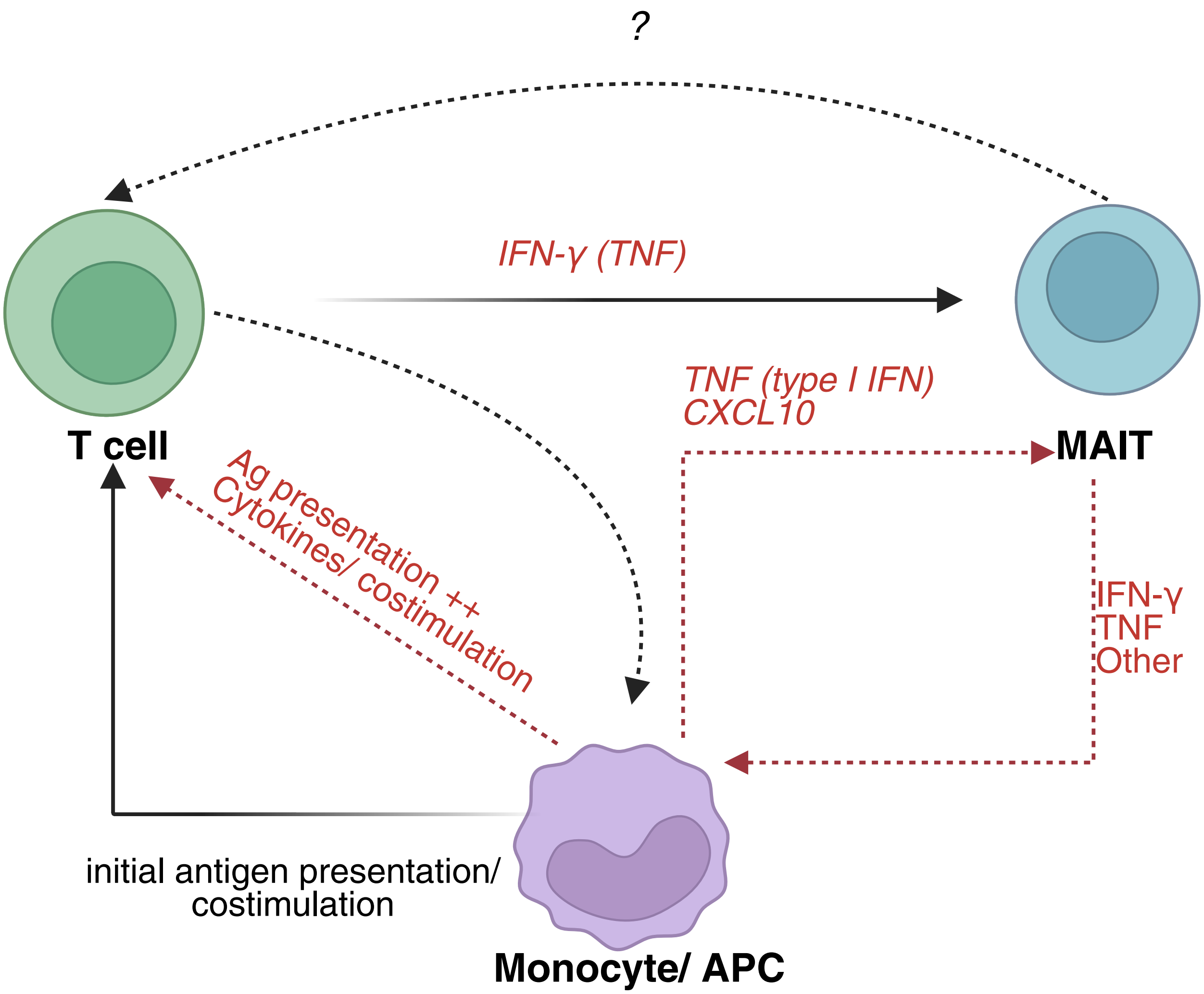

Figure S12. Model for MAIT cell crosstalk with conventional T cells and monocytes. Triggering of antigen-specific T cells at vaccine boost leads to release of IFN-γ and other cytokines which act on innate-like T cells such as MAITs, as well as myeloid cells such as monocytes (adaptive-to-innate pathway). Feed-forward loops within innate-like T cell populations and between T cells and monocytes may serve to amplify cytokine release.

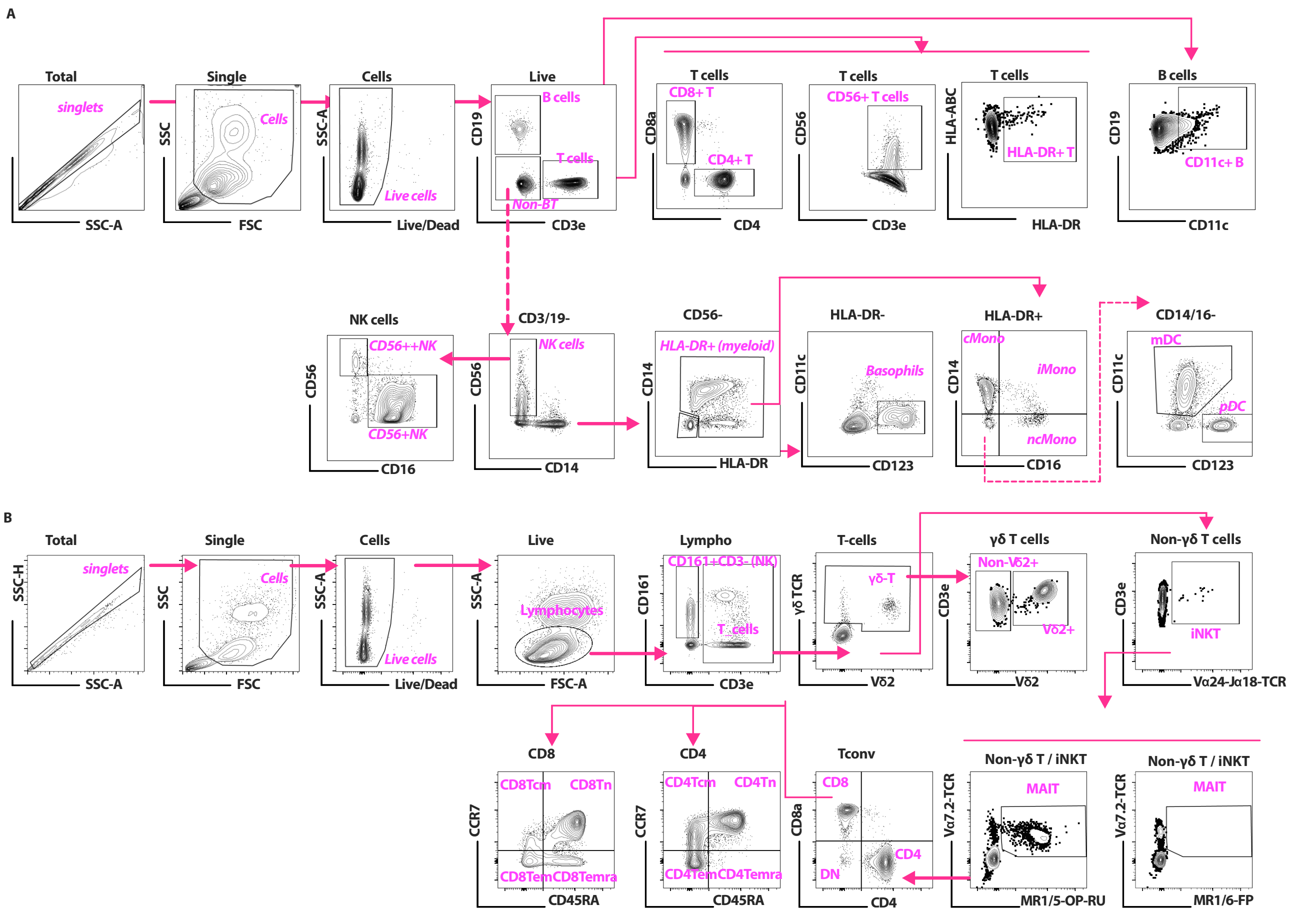

Figure S13. Gating strategies for cellular phenotyping. (A) Representative gating scheme for identification of major innate immune cell subsets in PBMCs, including expression of HLA-A/B/C, HLA-DR, and TNFR2. **(B)** Representative gating scheme for identification of major innate lymphocyte populations, including CD161^{+}CD3^{-} NK cells. MAIT cells defined as CD3^{+}, MR1/5-OP-RU^{+}, TCR-Vα7.2^{+} lymphocytes; MR1/6-FP tetramer used for gating. Abbreviations: cMono, classical monocytes (CD14^{+}CD16^{-}); iMono, intermediate monocytes (CD14^{+}CD16^{+}); ncMono, non-classical monocytes (CD14^{-}CD16^{+}); mDC, myeloid dendritic cells; DN, double negative; iNKT, invariant natural killer T cells; MAIT, mucosal-associated invariant T cells; Tn, naïve T cells (CCR7^{+}CD45RA^{+}); Tcm, central memory T cells (CCR7^{+}CD45RA^{-}); Tem, effector memory T cells (CCR7^{-}CD45RA^{-}); Temra, RA^{+} effector memory T cells (CCR7^{-}CD45RA^{+}). Antibodies used for flow cytometry listed in **Table S7**.

Supplementary Tables

	Vaccine Regimen				
	**Overall, N = 56**	**BNT162b2-Short, N = 17**	**BNT162b2-Long, N = 19**	**ChAdOx1-S, N = 20**	**p-value**
Boost interval, days	66 (28- 77) [17, 120]	21 (21- 25) [17, 30]	70 (62- 78) [42, 120]	76 (70- 79) [60, 98]	<0.001
Prior infection					>0.99
No	44 (79%)	13 (76%)	15 (79%)	16 (80%)	
Yes	12 (21%)	4 (24%)	4 (21%)	4 (20%)	
Age, years	35 (30-43) [21, 65]	37 (35-44) [30, 58]	33 (31-38) [24, 65]	28 (22-46) [21, 59]	0.031
Sex					0.19
F	36 (64%)	8 (47%)	13 (68%)	15 (75%)	
M	20 (36%)	9 (53%)	6 (32%)	5 (25%)	
BMI	23.4 (20.3-26.0) [18.6, 34.1]	23.5 (20.4-25.8) [19.0, 29.7]	24.5 (21.9-27.3) [18.6, 34.1]	23.1 (20.3-25.6) [19.0, 28.1]	0.63
Unknown	18	3	7	8	
Smoking					0.38
Never Smoked	52 (93%)	15 (88%)	17 (89%)	20 (100%)	
Past Smoker	4 (7.1%)	2 (12%)	2 (11%)	0 (0%)	
Ethnicity					0.81
East Asian	5 (8.9%)	2 (12%)	2 (11%)	1 (5.0%)	
Other	3 (5.4%)	2 (12%)	1 (5.3%)	0 (0%)	
South Asian	3 (5.4%)	1 (5.9%)	1 (5.3%)	1 (5.0%)	
White	45 (80%)	12 (71%)	15 (79%)	18 (90%)	

Table S1. Summary of healthcare worker cohort. Data are presented as median (IQR [Range]) or n (%). Prior infection was determined based on history of infection or presence of anti-spike binding antibodies pre-vaccination. BMI (body mass index). Comparisons for median

values for continuous data were performed using Kruskal-Wallis rank-sum test (interval, age, BMI). Comparisons for categorical data were performed using Pearson's Chi-squared test (sex), or Fisher's exact test (infection, smoking, ethnicity).

Variables	Odds Ratio	Std. Err.	z-value	p-value	95% Conf. Interval	Sig
ELISpot pre-boost	2.090	0.606	2.54	0.011	1.184-3.689	*
Vaccination prime regimen						
BNT, 28 days	0.694	0.250	-1.02	0.310	0.343-1.404	
ChAd, 84 days	0.462	0.171	-2.09	0.037	0.224-0.954	*
BNT, 84 days	0.282	0.123	-2.91	0.004	0.120-0.661	**
Age (years)	1.015	0.029	0.54	0.589	0.960-1.074	
Sex (Female)	1.376	0.368	1.19	0.232	0.815-2.325	
Constant	0.066	0.116	-1.54	0.124	0.002-2.104	

Mean dependent var	0.240	SD dependent var	0.428
Pseudo r-squared	0.078	Number of obs	342
Chi-square	29.468	Prob > chi2	0.000
Akaike crit. (AIC)	361.287	Bayesian crit. (BIC)	388.130

Table S2. Logistic regression analysis of factors associated with chills after BNT162b2 boost. Logistic regression of the probability of developing chills after a BNT162b2 booster. Variables include age, sex, pre-boost T cell IFN-γ ELISpot, and vaccine regimen (prime vaccine, interval). Variable references are “ChAd-BNT” with a 28 day interval for vaccination schedule, and “Male” for sex. Odds ratios (ORs) with 95% confidence intervals (CIs) are presented. *$p < 0.05$; **$p < 0.01$. ChAd, ChAdOx1-S; BNT, BNT162b2.

Variables	Odds Ratio	Std. Err.	z-value	p-value	95% Conf. Interval	Sig
ELISpot pre-boost	1.849	0.553	2.06	0.040	1.029-3.322	*
Vaccination prime regimen						
BNT, 28 days	0.501	0.182	-1.91	0.056	0.246-1.019	
ChAd, 84 days	0.772	0.284	-0.70	0.482	0.375-1.589	
BNT, 84 days	0.312	0.114	-3.18	0.001	0.153-0.640	**
Age (years)	1.020	0.026	0.79	0.431	0.971-1.072	
Sex (Female)	1.591	0.382	1.94	0.053	0.994-2.546	
Constant	0.335	0.537	-0.68	0.495	0.015-7.721	

Mean dependent var	0.629	SD dependent var	0.484
Pseudo r-squared	0.071	Number of obs	342
Chi-square	32.109	Prob > chi2	0.000
Akaike crit. (AIC)	433.104	Bayesian crit. (BIC)	459.947

Table S3. Logistic regression analysis of factors associated with fatigue after BNT162b2 boost. Logistic regression of the probability of developing fatigue after a BNT162b2 booster. Variables include age, sex, pre-boost T cell IFN-γ ELISpot, and vaccine regimen (prime vaccine, interval). Variable references are “ChAd-BNT” with a 28 day interval for vaccination schedule, and “Male” for sex. Odds ratios (ORs) with 95% confidence intervals (CIs) are presented. *$p < 0.05$; **$p < 0.01$. ChAd, ChAdOx1-S; BNT, BNT162b2.

Characteristic	Overall, N = 56	BNT162b2-Short N = 17	BNT162b2-Long N = 19	ChAdOx1-S N = 20
Prime	1 (0-2) [0, 154]	1 (0.5-9.5) [0, 154]	1 (0-4) [1, 98]	0 (0-41.5) [0, 107]
Boost	1 (0-2) [0, 11]	0 (0-1) [0, 3]	1 (0-1.75) [0, 11]	0.5 (0-2) [0, 6]

Table S4. Summary of interval between sample time and vaccine date. Data are presented as median (IQR) [Range].

	**Female**	**Male**	**Total**
Number (%)	26 (52)	24 (48)	50 (100)
Age (years)	60 (48-67)	62 (61-65)	62 (51-66)
Median days from second vaccine dose	160 (154-165)	155 (153-158)	157 (153-163)
BMI (kg/m^2)	34.2 (28.9-41.3)	29.8 (26.1-38.2)	32.0 (26.4-40.3)
Active smoker; n (%)	2 (8)	1 (4)	3 (6)
Former smoker; n (%)	6 (23)	9 (38)	15 (30)
Never smoked; n (%)	18 (69)	14 (58)	32 (64)
Hypertension; n (%)	12 (46)	11 (46)	23 (46)

Table S5. Summary of cohort for samples used for in vitro experiments (Dublin). Data are presented as median (IQR)**.**

Antibody	Clone	Supplier	Catalogue Number
Mouse IgG1 isotype control	MOPC-21	BioLegend	#400102
Mouse IgG2a isotype control	MOPC-173	BioLegend	#400202
Anti-MR1	26.5	BioLegend	#361102
Anti-MHC-I (HLA-A, B, C)	W6/32	BioLegend	#311402
Anti-MHC-II (HLA-DR, DP, DQ)	TÜ39	BioLegend	#361702
Anti-IFN-γR1 (CD119)	MAB6731	R&D	#92101
Adalimumab (anti-TNF)	-	AbbVie Inc	-

Table S6. Blocking and inhibitory antibodies used in stimulation assays.

Table S7. List of flow cytometry antibodies. (Excel file tab)

Table S8. List of TCR and Cytokine Genes. (Excel file)

Table S9. List of SARS-CoV-2 peptides used in this study. (Excel file tab)
